# Discovery of Nanosota-EB1 and -EB2 as Novel Nanobody Inhibitors Against Ebola Virus Infection

**DOI:** 10.1371/journal.ppat.1012817

**Published:** 2024-12-23

**Authors:** Fan Bu, Gang Ye, Kimberly Morsheimer, Alise Mendoza, Hailey Turner-Hubbard, Morgan Herbst, Benjamin Spiller, Brian E. Wadzinski, Brett Eaton, Manu Anantpadma, Ge Yang, Bin Liu, Robert Davey, Fang Li

**Affiliations:** 1 Department of Pharmacology, University of Minnesota Medical School, Minneapolis, Minnesota, United States of America; 2 Center for Emerging Viruses, University of Minnesota, Minneapolis, Minnesota, United States of America; 3 National Emerging Infectious Diseases Laboratories, Boston University, Boston, Massachusetts, United States of America; 4 Department of Virology, Immunology, and Microbiology, Boston University School of Medicine, Boston, Massachusetts, United States of America; 5 Department of Pharmacology, Vanderbilt University School of Medicine, Nashville, Tennessee, United States of America; 6 Integrated Research Facility at Fort Detrick, Division of Clinical Research, National Institute of Allergy and Infectious Diseases, National Institutes of Health, Frederick, Maryland, United States of America; 7 Hormel Institute, University of Minnesota, Austin, Minnesota, United States of America; University of Texas Medical Branch / Galveston National Laboratory, UNITED STATES OF AMERICA

## Abstract

The Ebola filovirus (EBOV) poses a serious threat to global health and national security. Nanobodies, a type of single-domain antibody, have demonstrated promising therapeutic potential. We identified two anti-EBOV nanobodies, Nanosota-EB1 and Nanosota-EB2, which specifically target the EBOV glycoprotein (GP). Cryo-EM and biochemical data revealed that Nanosota-EB1 binds to the glycan cap of GP1, preventing its protease cleavage, while Nanosota-EB2 binds to critical membrane-fusion elements in GP2, stabilizing it in the pre-fusion state. Nanosota-EB2 is a potent neutralizer of EBOV infection in vitro and offers excellent protection in a mouse model of EBOV challenge, while Nanosota-EB1 provides moderate neutralization and protection. Nanosota-EB1 and Nanosota-EB2 are the first nanobodies shown to inhibit authentic EBOV. Combined with our newly developed structure-guided in vitro evolution approach, they lay the foundation for nanobody-based therapies against EBOV and other viruses within the ebolavirus genus.

## Author summary

Ebola virus (EBOV) poses a serious global health threat due to its high fatality rate. Current antibody-based treatments face significant challenges, including reducing mortality, addressing viral persistence in some survivors, and ensuring affordability and accessibility. Furthermore, adapting existing human antibody therapies to combat EBOV-related viruses, such as Bundibugyo ebolavirus (BDBV) and Sudan ebolavirus (SUDV), remains difficult. Nanobodies, with their modular single-domain structure, present a promising solution to these challenges. In this study, we identified two anti-EBOV nanobodies, Nanosota-EB1 and Nanosota-EB2, which specifically target the EBOV GP protein. Cryo-EM and biochemical analyses revealed that Nanosota-EB1 binds to the glycan cap of GP1, preventing protease cleavage essential for viral infection. In contrast, Nanosota-EB2 binds to GP2, stabilizing it in the pre-fusion state and blocking its ability to mediate fusion between viral and host membranes. Nanosota-EB2 demonstrates strong neutralizing activity against EBOV infection in vitro and provides robust protection in a mouse model of EBOV challenge, while Nanosota-EB1 offers moderate neutralization and protection. As the first nanobodies shown to be effective against authentic EBOV, Nanosota-EB1 and Nanosota-EB2, together with our recently developed nanobody evolution strategy, establish a foundation for nanobody-based therapies targeting EBOV and related filoviruses.

## Introduction

The Ebola virus (EBOV), a member of the ebolavirus genus in the filovirus family, poses a serious threat to global health and national security due to its high fatality rate. During the unprecedented outbreak in West Africa from 2014 to 2016, there were 28,652 infections with a case fatality rate of ~ 40% [1,2]. Smaller outbreaks of EBOV and related filoviruses have occurred periodically [3]. Although bats have been suggested as a potential reservoir, intact EBOV has yet to be isolated from bats [4]. Additionally, EBOV can remain dormant in the human body for years before reemerging to cause new infections [5]. Given the long-term coexistence of EBOV with humans, developing effective and accessible treatment strategies is a critical priority.

The GP protein of EBOV guides viral entry into human cells and is a primary target for neutralizing antibodies [6]. GP is presented on the virus surface as a homotrimer, composed of three copies of the receptor-binding subunit GP1 and a trimeric stalk formed by the membrane-fusion subunit GP2 (S1A Fig), which also anchors the trimer in the viral membrane via a transmembrane region [7]. GP1 contains a receptor-binding site (RBS) that is concealed by a glycan cap and a mucin-like domain (MLD) [6,8]. GP2 features a fusion peptide and two heptad repeat regions (HR1 and HR2) [9]. During molecular maturation, GP is cleaved by the human protease furin at the GP1/GP2 boundary, but GP1 and GP2 remain associated in a metastable "pre-fusion" state. During cell entry, GP1 interacts with cell-surface factors to facilitate viral attachment to host cells, after which the virus is taken up by endocytosis [10]. Inside the endosomes, cathepsins remove the glycan cap and MLD, exposing the RBS [11]. The RBS then binds to its host receptor Niemann-Pick C1 (NPC1) on the endosomal membranes [12,13]. Subsequently, GP2 transitions to its "post-fusion" state, the lowest-energy conformation, where HR1 and HR2 form a six-helix bundle, allowing the fusion peptide to merge the viral and host membranes [14,15]. An effective anti-EBOV antibody therapy may inhibit protease cleavage, block receptor binding, or prevent the structural transition of EBOV GP. A challenge in anti-EBOV antibody therapy is that EBOV secretes large amounts of sGP, a secreted form of GP that includes most regions of GP1 but none of GP2, as a decoy to divert GP1-targeting antibodies [16]. The primary transcript of the GP gene is sGP mRNA (~75%). Occasionally, the viral polymerase pauses near the sGP stop codon, causing a frameshift that leads to the production of full-length GP mRNA (~25%) [17]. Effective anti-EBOV antibody therapy must block GP-guided viral entry and resist sGP diversion simultaneously.

Currently, two FDA-approved human antibody drugs target EBOV GP [18,19]. One of these, Ebanga, is a single antibody that targets the RBS and blocks receptor binding, though it may be susceptible to viral escape due to epitope mutations. Additionally, there has been a case of acute Ebola virus disease (EVD) relapse in a survivor from an earlier EBOV outbreak who had previously been treated with Ebanga [20]. In contrast, Inmazeb, a cocktail of three antibodies that control infection by targeting three distinct epitopes, makes viral escape more difficult [21]. One of the three antibodies targets the RBS to block receptor binding, another targets the glycan cap and inhibits protease cleavage of the glycan cap, and the third targets GP2, although its mechanism of action has not been fully characterized. Both of these approved antibody drugs have reduced the fatality rate of EBOV infections to around 35%, including in some late-stage cases [18,19]. Furthermore, some EVD survivors show evidence of viral persistence, where the large size of conventional antibodies may hinder access to immune sanctuary sites [21–23]. Moreover, the high costs of production, transport, and storage for human antibodies, coupled with their injection-only administration, present significant barriers. Currently, no FDA-approved drugs or vaccines exist for other members of the ebolavirus genus, such as Bundibugyo ebolavirus (BDBV) and Sudan ebolavirus (SUDV), which continue to pose serious threats to global health and national security [24,25]. Adapting anti-EBOV human antibodies to combat related filoviruses can be challenging. Thus, there is an urgent need to develop potent, small, cost-effective, and broadly accessible therapies for EBOV infections, with potential applicability to other filoviruses.

Nanobodies are single-domain antibodies derived from the heavy-chain-only antibodies produced by camelid animals and sharks [26,27]. Their single-domain structure offers numerous advantages over conventional antibodies in antiviral applications. For instance, nanobodies exhibit excellent epitope accessibility and tissue permeability [28,29], which enhance their antiviral efficacy and may benefit EVD survivors with viral persistence. Moreover, nanobodies can be produced in high yields, demonstrate good in vitro thermostability, and are cost-effective to manufacture, transport, and store [30]. They can also be administered intranasally [31,32], making them a promising option for needle-free therapies. Additionally, due to their high homology to human germline antibodies, nanobodies show minimal toxicity and immunogenicity in humans [28,29]. In 2019, the first nanobody-based therapeutic was FDA-approved to treat a blood clotting disorder [33]. During the COVID-19 pandemic, we developed a series of nanobody inhibitors, known as the Nanosota series, which demonstrated exceptional potency and broad-spectrum activity against SARS-CoV-2 variants [30,32,34,35]. Importantly, we recently developed a novel structure-guided in vitro evolution approach for nanobodies [36], allowing rapid adaptation of nanobodies to target different viral variants. Previously, only one study reported a nanobody targeting pseudoviruses packaged with the EBOV GP protein [37]. To date, no nanobody inhibitors have been developed or evaluated against authentic EBOV either in vitro or in vivo.

In this study, we identified two nanobodies, Nanosota-EB1 and Nanosota-EB2, which bind to distinct epitopes on the EBOV GP protein. Using cryo-EM and biochemical assays, we investigated their mechanisms of action and assessed their anti-EBOV efficacy both in vitro and in a mouse model. These nanobodies represent a promising, cost-effective, and accessible therapeutic option for EBOV infections. Combined with our recently developed nanobody evolution approach, they provide a foundation for nanobody-based therapies targeting EBOV and related filoviruses.

## Results

### Discovery and in vitro characterization of Nanosota-EB1 and -EB2

To identify nanobodies targeting EBOV, we produced a recombinant version of the EBOV GP ectodomain lacking the mucin-like domain (MLD). The MLD, located at the top of each GP monomer, shields the receptor-binding site (RBS) from immune recognition. Its removal does not affect the overall GP structure but enhances GP ectodomain expression [38]. This modified GP was named GP-ΔM (S1B Fig). An alpaca was immunized with this protein, and peripheral blood mononuclear cells were collected to construct a phage display library containing the alpaca’s nanobodies. Using GP-ΔM as bait, we screened the library and identified six nanobodies that bound to GP-ΔM. Among these, two nanobodies, named Nanosota-EB1 and Nanosota-EB2 (abbreviated as EB1 and EB2, respectively), exhibited the highest affinity for GP-ΔM based on initial ELISA results. We expressed and purified the His-tagged nanobodies (EB1-His and EB2-His) from bacteria, achieving expression yields of over 20 mg/L of medium for each. Moreover, we expressed and purified the Fc-tagged versions (EB1-Fc and EB2-Fc) from mammalian cells, with expression yields exceeding 50 mg/L of medium for each. These purified nanobodies were subsequently used for functional and structural characterization.

**Fig 1:**
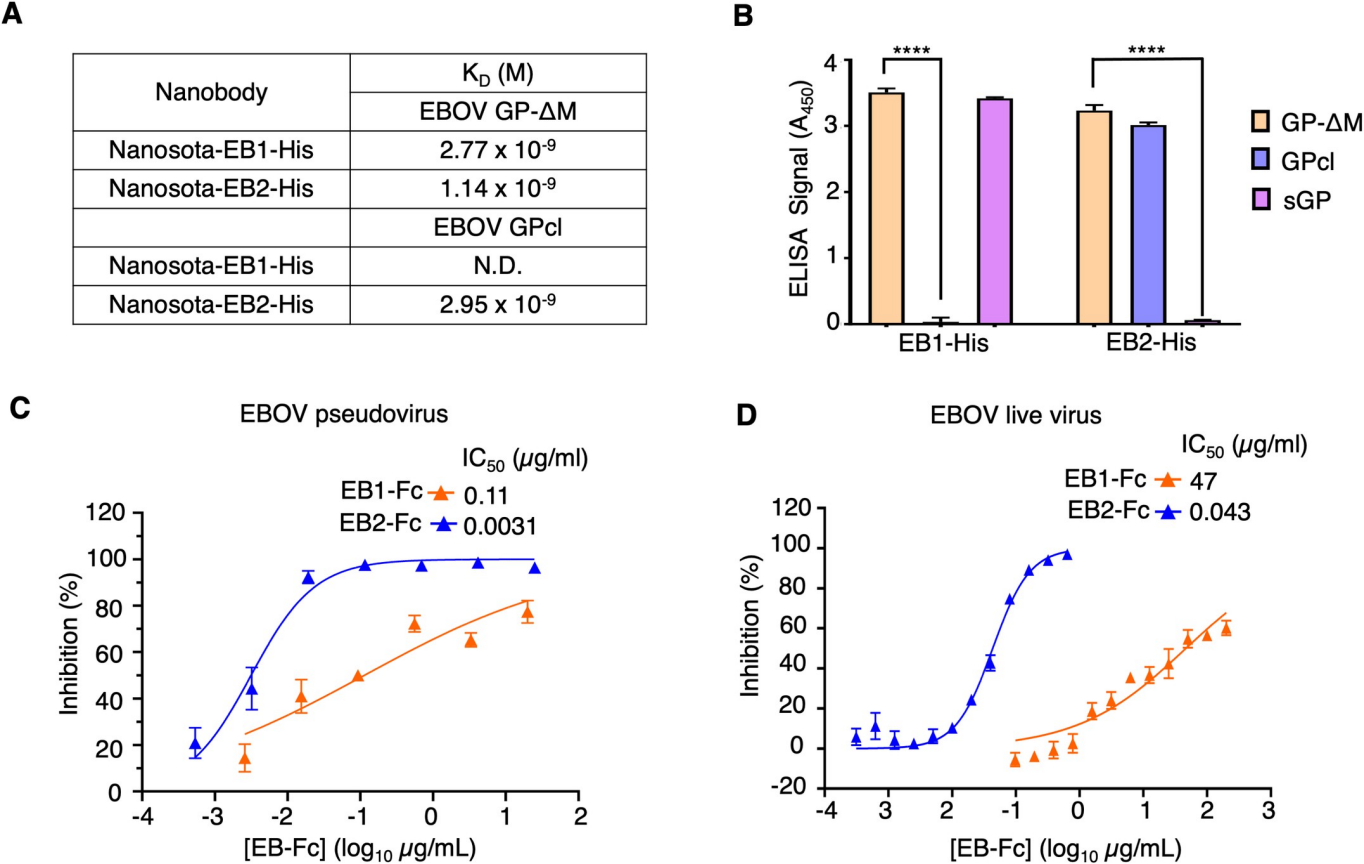
In Vitro Characterization of Two Novel Anti-EBOV Nanobodies, Nanosota-EB1 and -EB2. **(A)** Binding affinities between His-tagged nanobodies and two versions of EBOV GP proteins (GP-ΔM and GPcl; see S1B Fig for their definitions) were measured by surface plasmon resonance (SPR). N.D., binding not detected. **(B)** Binding interactions between His-tagged nanobodies and three versions of the EBOV GP proteins (GP-ΔM, GPcl and sGP; see S1B Fig for their definitions) were evaluated by ELISA. A_450_: absorbances at 450 nm. Data are presented as mean ± SEM (n = 3). An unpaired two-tailed Student’s *t*-test was used to analyze the statistical differences between the indicated groups, with results indicated above each bar. ****P < 0.0001. **(C)** Efficacy of Fc-tagged nanobodies in neutralizing EBOV pseudoviruses. Retroviruses pseudotyped with full-length EBOV GP were used to infect Huh7 cells in the presence of Fc-tagged Nansota-EB1 or -EB2 at different concentrations. The efficacy of each nanobody against EBOV pseudoviruses was expressed as the concentration required to neutralize pseudovirus entry by 50% (IC_50_). Error bars represent SEM (n = 3). **(D)** Efficacy of Fc-tagged nanobodies in neutralizing authentic EBOV infection. Authentic EBOV was used to infect Huh7 cells in the presence of Fc-tagged Nanosota-EB1 or -EB2 at different concentrations. The efficacy of each nanobody against authentic EBOV infection was expressed as the concentration required to neutralize EBOV infection by 50% (IC_50_). Error bars represent SEM (n = 3). Since Nanosota-EB1-Fc could not fully block viral entry at any of the tested concentrations, the IC_50_ values for Nanosota-EB1-Fc are estimations.

We characterized the binding of EB1 and EB2 to the EBOV GP protein. First, we used surface plasmon resonance (SPR) to evaluate the binding affinity of the His-tagged nanobodies to GP-ΔM. Both EB1-His and EB2-His demonstrated high-affinity binding to GP-ΔM, with dissociation constants (K_d_) in the nanomolar range (Figs 1A and S2). Second, SPR was performed to measure the binding affinity of the nanobodies to a further cleaved GP ectodomain (GPcl), which lacks both the MLD and the glycan cap (S1B Fig). The results showed that EB1-His does not bind to GPcl, whereas EB2-His retains high-affinity binding with a K_d_ value in the nanomolar range (Figs 1A and S2), suggesting that EB1 interacts with the glycan cap, unlike EB2. Additionally, we used ELISA to assess the binding of the His-tagged nanobodies to sGP, the secreted form of GP that includes most of GP1 but none of GP2 (S1B Fig), comparing it to GP-ΔM and GPcl. The results revealed that EB1-His binds to sGP, while EB2-His does not (Fig 1B). Taken together, these findings indicate that EB1 targets the glycan cap of GP1 and sGP, whereas EB2 does not, despite both nanobodies exhibiting strong binding to GP-ΔM.

Next, we evaluated the efficacy of EB1 and EB2 in neutralizing EBOV entry in vitro. Lentiviruses pseudotyped with the full-length EBOV GP (including the glycan cap and MLD), referred to as EBOV pseudoviruses, were used to measure their entry into human cells in the presence of the nanobodies. Both His-tagged and Fc-tagged nanobodies were tested. The results showed that EB1-Fc and EB2-Fc neutralized EBOV pseudovirus entry with moderate and high potency, achieving IC_50_ values of 110 ng/ml and 3.1 ng/ml, respectively (Fig 1C). Furthermore, the Fc-tagged nanobodies exhibited stronger neutralization than their His-tagged counterparts (S3 Fig), likely due to increased valency provided by the Fc tag. We also tested the Fc-tagged nanobodies against authentic EBOV infection in human cells. EB1-Fc and EB2-Fc neutralized authentic EBOV infection with low and high potency, achieving IC_50_ values of 47 μg/ml and 43 ng/ml, respectively (Fig 1D). The ~1000-fold reduction in EB1-Fc’s neutralizing efficacy against authentic EBOV infection assay compared to pseudovirus entry assay is likely due to the large amounts of sGP secreted by EBOV-infected cells. In contrast, the neutralizing efficacy of EB2-Fc decreased by only ~10-fold in the same comparison, reflecting a calibration difference between the pseudovirus and authentic EBOV neutralization assays, as EB2 does not interact with sGP. Overall, these findings confirm that EB1 is a moderate inhibitor of EBOV entry, with its activity further diminished against authentic EBOV due to the effects of sGP. Conversely, EB2 is a highly effective inhibitor of both EBOV pseudovirus entry and authentic EBOV infection, with its efficacy unaffected by sGP.

### Structural basis for anti-EBOV functions of Nanosota-EB1 and -EB2

**Fig 2:**
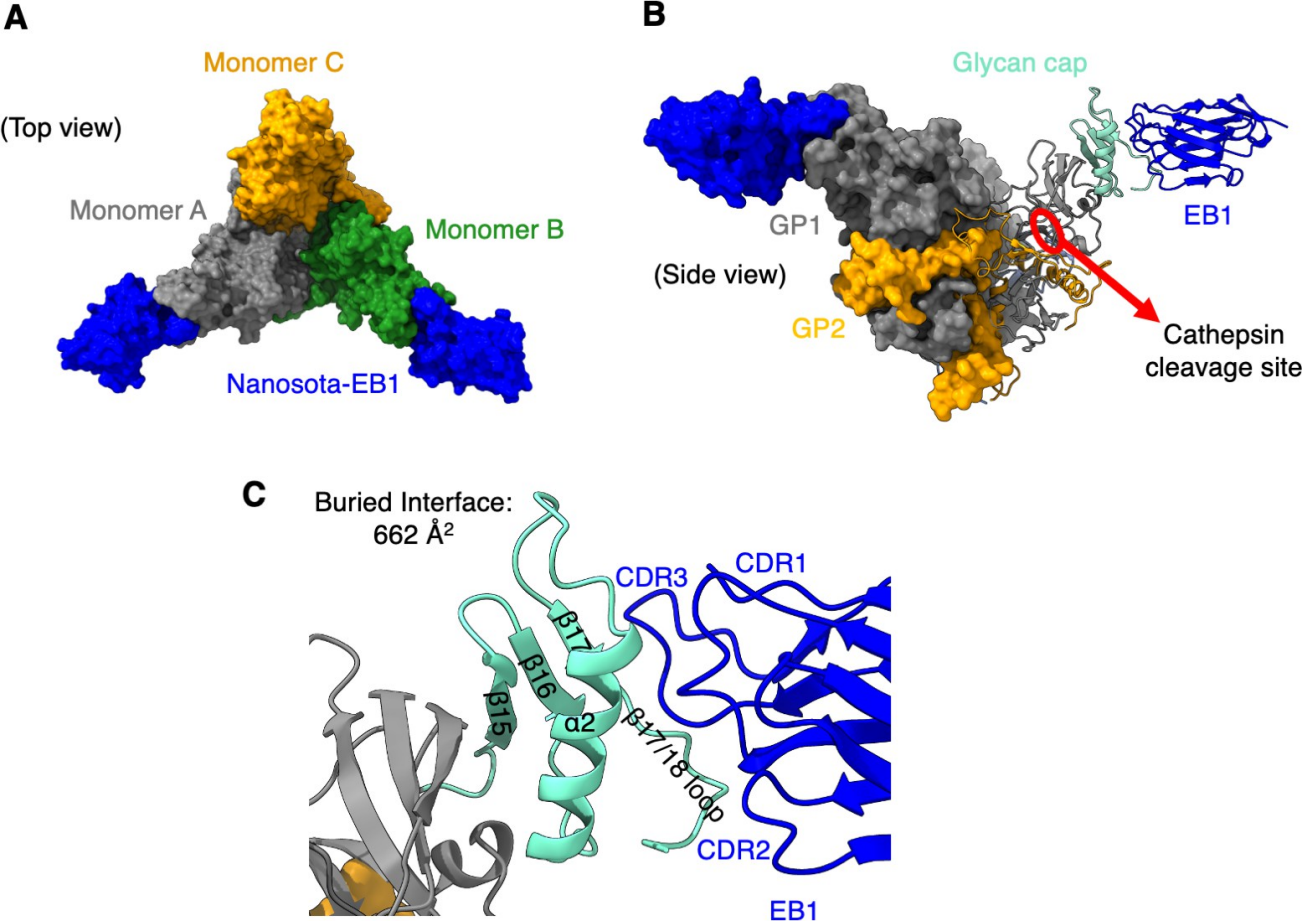
Structural Basis for the Anti-EBOV Functions of Nanosota-EB1. **(A)** Cryo-EM structure of EBOV GP-ΔM complexed with Nanosota-EB1 (top view; surface presentation). The three subunits of EBOV GP-ΔM are colored orange, gray, and green, respectively. Nanosota-EB1 is shown in blue. The trimeric GP-ΔM is bound by two Nanosota-EB1 molecules. **(B)** Cryo-EM structure of EBOV GP-ΔM complexed with Nanosota-EB1 (side view). The overall structure is shown in surface presentation, with one GP subunit and one Nanosota-EB1 molecule shown in cartoon presentation. Nanosota-EB1 binds to the glycan cap of EBOV GP. The glycan cap is colored cyan. The cathepsin cleavage site near the glycan cap is marked by a red circle. **(C)** The binding interface between Nanosota-EB1 and the glycan cap. Nanosota-EB1 binds to the β17 strand of the glycan cap, displacing the β18 strand and pushing it aside to form a loop.

To investigate the structural basis of the nanobodies’ inhibition of EBOV entry, we determined the cryo-EM structures of EBOV GP-ΔM in complex with EB1-His and EB2-His, respectively (S4–S6 Figs and S1 Table). The structure of GP-ΔM in complex with EB1 revealed that the trimeric GP-ΔM is engaged by two EB1 molecules (Fig 2A and 2B). EB1 binds to the top of the glycan cap, burying 662 Å^2^ of surface area at the interface (Fig 2C). In the absence of EB1, the glycan cap density is not visible, indicating that it is disordered (S7A Fig). However, upon EB1 binding, the glycan cap becomes ordered (S7A Fig). The human antibody REGN-3470 targets the glycan cap by binding to the outermost β-18 strand (S7B Fig). Unlike REGN-3470, EB1 displaces the β-18 strand, pushing it aside and exclusively interacting with the inner β-17 strand (S7C Fig). Another human antibody, EBOV-548, also targets the glycan cap by displacing the β-18 strand and binding to the inner β-17 strand. However, EB1 and EBOV-548 bind to the glycan cap from different orientations (S7D Fig). These structural findings suggest that, similar to glycan cap-targeting human antibodies [39], EB1 may inhibit EBOV entry by stabilizing the glycan cap, potentially preventing its cleavage by cathepsins.

**Fig 3:**
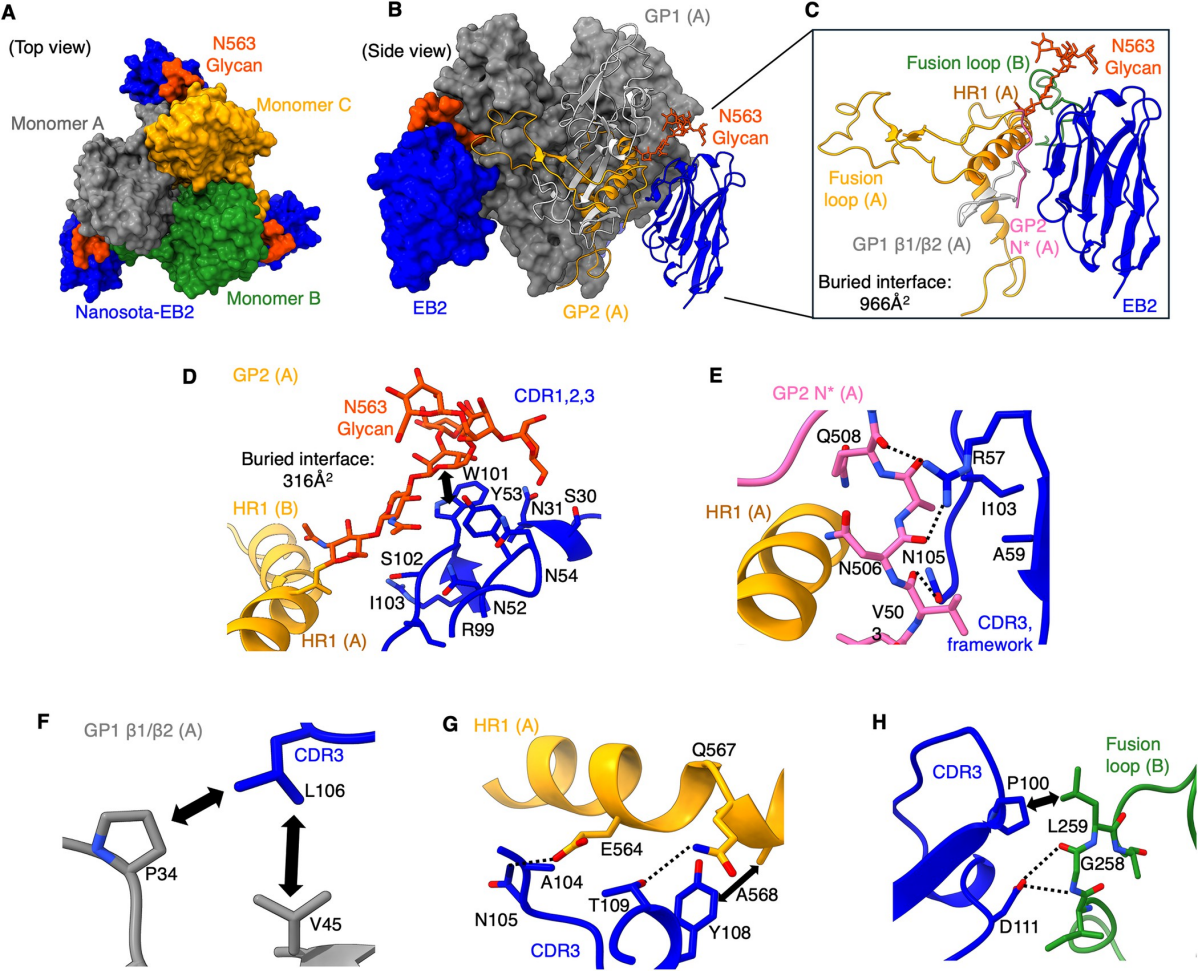
Structural Basis for the Anti-EBOV Functions of Nanosota-EB2. **(A)** Cryo-EM structure of EBOV GP-ΔM complexed with Nanosota-EB2 (top view; surface presentation). The three subunits of EBOV GP-ΔM are colored orange, gray, and green, respectively. Nanosota-EB2 is shown in blue. The N563 glycan involved in binding Nanosota-EB2 is shown in red. The trimeric GP-ΔM is bound by three Nanosota-EB2 molecules. **(B)** Cryo-EM structure of EBOV GP-ΔM complexed with Nanosota-EB2 (side view). The overall structure is shown in surface presentation, with one Nanosota-EB2 molecule and several membrane-fusion elements in GP shown in cartoon presentation, and the N563 glycan shown in sticks. **(C)** The binding interface between Nanosota-EB2 and GP. Nanosota-EB2 recognizes quaternary epitopes, including HR1, fusion loop, N-terminus of GP2, and β1/β2 strands of GP1. **(D)-(H)** Detailed interactions between Nanosota-EB2 and N563 glycan, HR1, fusion loop, N-terminus of GP2, and β1/β2 strands of GP1, respectively. Dotted lines indicate hydrogen bonds. Double arrows indicate hydrophobic interactions.

The structure of GP-ΔM in complex with EB2-His revealed that each trimeric GP-ΔM molecule is bound by three EB2 molecules (Fig 3A). Each EB2 binds to relatively conserved quaternary epitopes on GP, including HR1, the fusion loop, the N-terminus of GP2, and the β1/β2 strands of GP1, forming a large buried interface of 966 Å^2^ (Fig 3B and 3C). All three complementarity-determining regions (CDRs) of EB2 strongly interact with an N-linked glycan on Asn563 (N563 glycan) of HR1, which is critical for GP2’s stability and membrane fusion function, burying 316 Å^2^ at the interface (Fig 3D). In the absence of EB2, the N563 glycan is less ordered, with fewer sugar rings visible (S8 Fig). Similarly, while the human antibody REGN-3479 also binds to the N563 glycan, its stabilization of the glycan is less pronounced, as evidenced by fewer visible sugar rings compared to EB2 (S8 Fig) [39]. EB2 stabilizes the glycan through strong hydrophobic interactions and hydrogen bonding (Fig 3D), pulling the glycan closer to itself (S8 Fig). This results in a significant positional shift of the N563 glycan, a phenomenon observed for the first time. Additionally, the CDR3 of EB2 engages with the N-terminus of GP2 (Fig 3E). In the absence of EB2, the residues at GP2’s N-terminus are flexible, with only a few residues visible. EB2 secures the N-terminus through strong hydrogen bonds, enhancing its visibility and locking it in place. The CDR3 also interacts with HR1 via hydrophobic interactions and hydrogen bonds (Fig 3F) and binds to the β1/β2 strands of GP1 through hydrophobic interactions (Fig 3G). Notably, EB2 forms interactions not only with a single GP subunit but also bridges two distinct GP subunits. For example, CDR3 of EB2 interacts with the fusion loop from a different GP subunit through hydrophobic interactions and hydrogen bonds (Fig 3H). These unique and robust interactions between EB2 and multiple GP epitopes suggest two mechanisms by which EB2 inhibits EBOV entry. First, EB2 stabilizes the N563 glycan and the N-terminus of GP2 while bridging two GP2 subunits and connecting GP1 and GP2, thereby enhancing the stability of the pre-fusion GP. Second, EB2 engages key membrane-fusion elements, including HR1 and the fusion peptide, locking them in their pre-fusion conformations.

**Fig 4:**
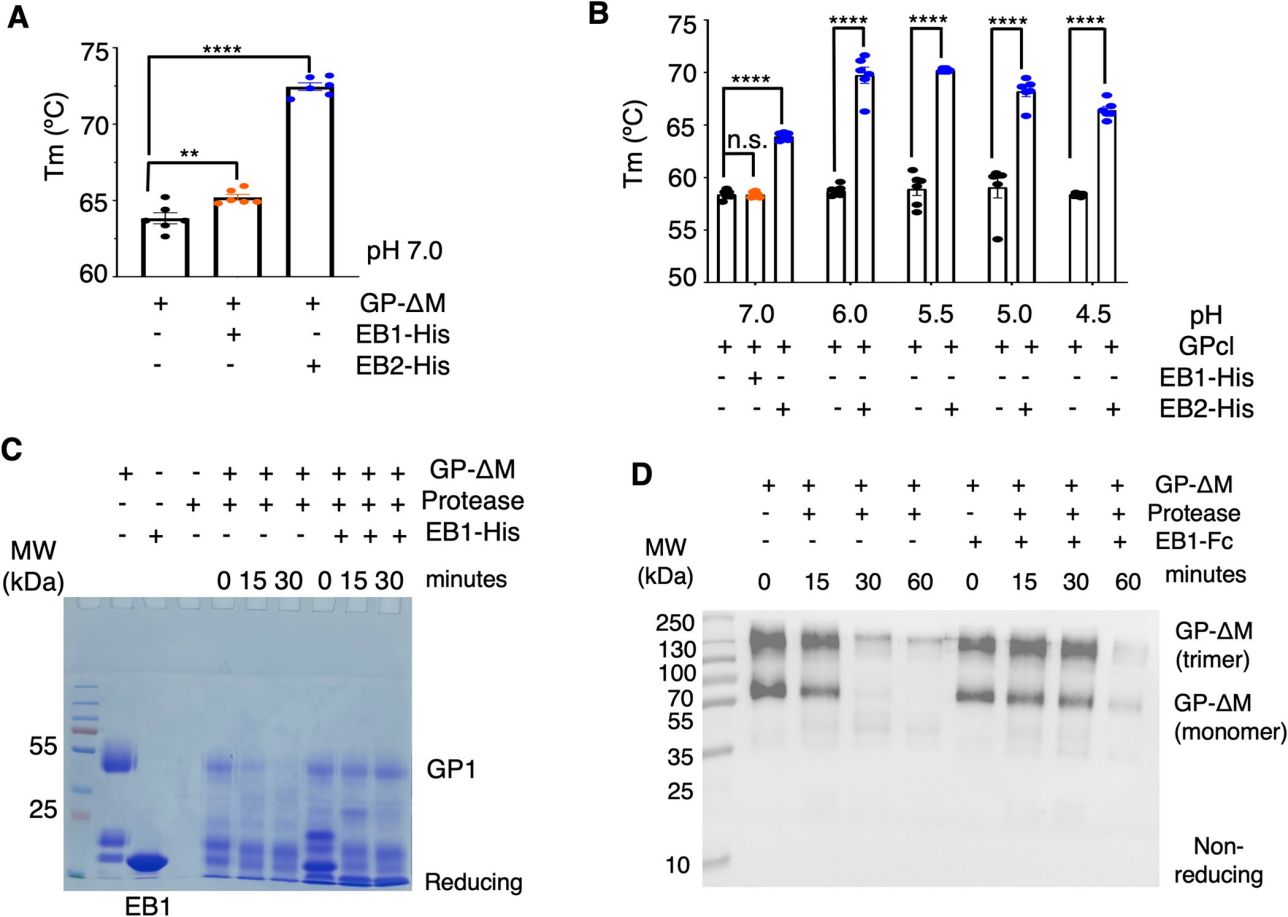
Biochemical Mechanisms for the Anti-EBOV Functions of Nanosota-EB1 and -EB2. **(A)** Differential scanning fluorimetry (DSF) assay for assessing the impact of nanobodies on the thermal stability of EBOV GP-ΔM. His-tagged Nanosota-EB1 and -EB2 slightly and significantly increased the thermostability of EBOV GP-ΔM, respectively. Comparisons of the Tm values for GP-ΔM in the absence or presence of the nanobodies were performed using an unpaired two-tailed Student’s *t*-test. Error bars represent SEM (n = 6). **p<0.01, ****p<0.0001. **(B)** DSF assay for assessing the impact of His-tagged Nanosota-EB2 on the thermal stability of EBOV GPcl at lower pHs. Nanosota-EB2 significantly increased the thermostability of EBOV GPcl at low pH. Comparisons of the Tm values for GPcl in the absence or presence of Nanosota-EB2 were performed using an unpaired two-tailed Student’s *t*-test. Error bars represent SEM (n = 6). ****p<0.0001. n.s.: not significant. Note that this experiment could not be conducted for Nanosota-EB1 because Nanosota-EB1 does not bind to EBOV GPcl. **(C)** Glycan cap cleavage assay to evaluate the effect of Nanosota-EB1 on the protease sensitivity of the glycan cap, using SDS-PAGE under reducing conditions and Coomassie blue staining. Nanosota-EB1 presence slowed the thermolysin L cleavage of GP-ΔM. **(D)** Glycan cap cleavage assay to evaluate the effect of Nanosota-EB1 on the protease sensitivity of the glycan cap, using Western blot to detect the His tag on GP-ΔM under non-reducing conditions. Nanosota-EB1 presence again slowed thermolysin L cleavage of GP-ΔM. Each of the above experiments was performed three times, yielding consistent results.

To validate the structural data, we conducted two biochemical assays. First, we examined how each nanobody affects the thermostability of GP-ΔM and GPcl by measuring the thermal shifts induced by EB1-His and EB2-His (Figs 4A, 4B and S9). At neutral pH, EB1 and EB2, both of which bind to GP-ΔM, increased its thermostability slightly (by 1°C) and significantly (by 6°C), respectively (Fig 4A). At the same pH, EB2, which binds to GPcl, significantly enhanced GPcl’s thermostability (Fig 4B), whereas EB1, which does not bind to GPcl, had no significant effect. At acidic pH levels (pH 4.5–6), which are physiologically relevant to endosomes, EB2 significantly increased GPcl’s thermostability by 11°C (Fig 4B). This suggests that EB2 strongly binds to and locks the prefusion GPcl even after cathepsin-mediated glycan cap cleavage in the endosomes. As a result, EB2 binding increases the energy barrier for GPcl to transition from its prefusion to post-fusion state, thereby blocking EBOV cell entry. This thermostability assay, the first conducted for GP-targeting antibodies/nanobodies, indicates that REGN-3479, which shares an overlapping epitope with EB2, may utilize a similar neutralization mechanism. Second, we investigated the effect of each nanobody on the protease sensitivity of GP-ΔM (Figs 4, S10 and S11). Previous studies have demonstrated that the bacterial protease thermolysin mimics endosomal proteases by cleaving the glycan cap and mucin-like domain from GP1 [40, 41]. GP-ΔM was treated with thermolysin L in the presence or absence of EB1 and EB2, and the glycan cap cleavage rate was monitored. Reducing SDS-PAGE analysis showed that the GP1 band disappeared completely after thermolysin L treatment in the absence of EB1 but remained nearly intact in its presence (Fig 4C). Similarly, non-reducing Western blot analysis revealed that the GP-ΔM band faded after thermolysin L treatment without EB1 but persisted longer when EB1 was present (Fig 4D and S11A). These findings indicate that EB1 delays protease-mediated glycan cap cleavage, whereas this effect is less pronounced with EB2 (S10 and S11B Figs). This observation aligns with previous research on glycan cap-binding human antibodies [39]. In summary, EB1 reduces GP’s protease sensitivity, while EB2 stabilizes GP, supporting the findings from our structural analysis.

**Fig 5:**
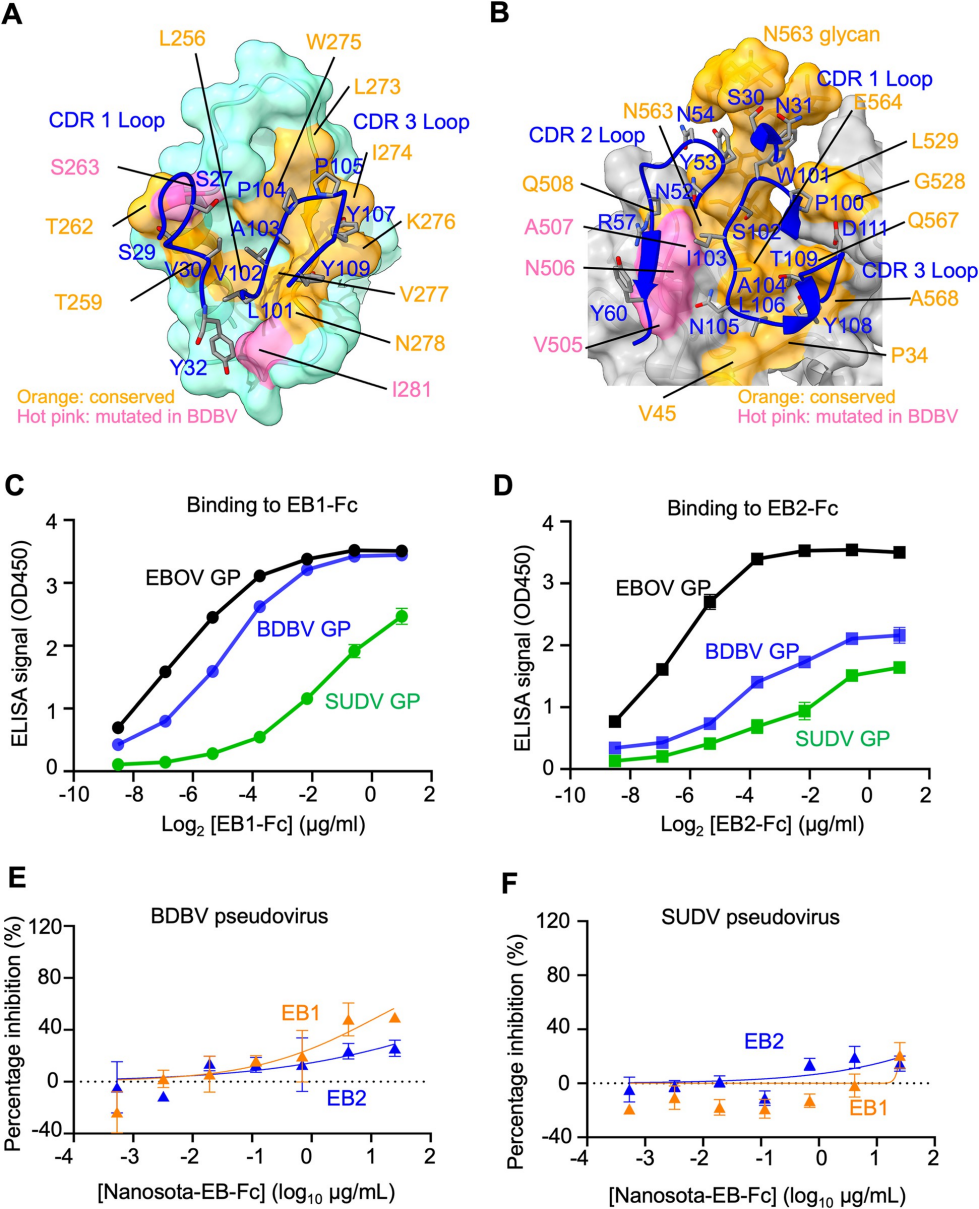
Footprints of Nanosota-EB1 and Nanosota-EB2 on EBOV GP and Their Binding to the GP of EBOV-Related Ebolaviruses. **(A)** Footprint of Nanosota-EB1 on the glycan cap of EBOV GP (surface representation). **(B)** Footprint of Nanosota-EB2 on the quaternary epitopes of EBOV GP (surface representation). Nanobody residues are labeled in blue. Nanobody-contacting residues on EBOV GP are categorized as follows: residues conserved between EBOV and BDBV or SUDV are marked in orange, while those differing between EBOV and BDBV or SUDV are highlighted in pink. **(C)** ELISA results showing the binding interactions between Nanosota-EB1-Fc and GP-ΔM from EBOV, BDBV, and SUDV. **(D)** ELISA results showing the binding interactions between Nanosota-EB2-Fc and GP-ΔM from EBOV, BDBV, and SUDV. **(E)** Neutralization efficacy of Fc-tagged nanobodies against BDBV pseudoviruses. **(F)** Neutralization efficacy of Fc-tagged nanobodies against SUDV pseudoviruses.

To evaluate the anti-ebolavirus spectrum of EB1 and EB2, we analyzed their specific interactions with EBOV GP. Among the EBOV residues directly interacting with EB1, two differ from those in BDBV, and five differ from those in SUDV (Fig 5A and S2 Table). Similarly, among the EBOV residues directly interacting with EB2, three differ from those in BDBV, and four differ from those in SUDV (Fig 5B and S3 Table). These differences suggest that neither EB1 nor EB2 serves as a strong cross-inhibitor of BDBV or SUDV. To validate the structural data, we conducted two biochemical assays. ELISA results demonstrated that EB1 binds less strongly to BDBV GP-ΔM compared to EBOV GP-ΔM and does not bind to SUDV GP-ΔM (Fig 5C), whereas EB2 fails to bind GP-ΔM from either BDBV or SUDV (Fig 5D). Pseudovirus entry assays further revealed that EB1 weakly neutralizes BDBV pseudoviruses but does not neutralize SUDV pseudoviruses (Fig 5E), while EB2 does not neutralize pseudoviruses from either BDBV or SUDV (Fig 5F). Thus, neither EB1 nor EB2 acts as a potent inhibitor of BDBV or SUDV. However, using our newly developed structure-guided in vitro evolution approach [36], EB1 and EB2 can potentially be engineered into pan-ebolavirus nanobody therapeutics.

### In vivo efficacy of Nanosota-EB1, -EB2, and their cocktail for treatment of disease

We assessed the therapeutic efficacy of Fc-tagged constructs—EB1-Fc, EB2-Fc, and their combination—in treating Ebola virus disease using a mouse model. Previous studies have shown that Fc-tagged nanobodies offer several advantages over His-tagged nanobodies as antiviral therapeutic candidates. These advantages include increased valency and enhanced neutralization potency, as well as a significantly longer in vivo half-life due to their size exceeding the kidney clearance threshold [30]. Despite this, Fc-tagged nanobodies are still only half the size of human antibodies. Importantly, they maintain a single-domain structure for target binding, exhibit high in vitro stability [30], and have the potential for intranasal administration [32]. The interferon-α/β receptor knockout (IFNAR) mouse model has been widely validated as a robust system for evaluating GP-targeting antibodies, as it reliably mimics the disease progression observed in humans infected with wild-type EBOV [42,43]. In this study, we investigated the anti-EBOV efficacy of EB1-Fc and EB2-Fc in the IFNAR mouse model.

**Fig 6:**
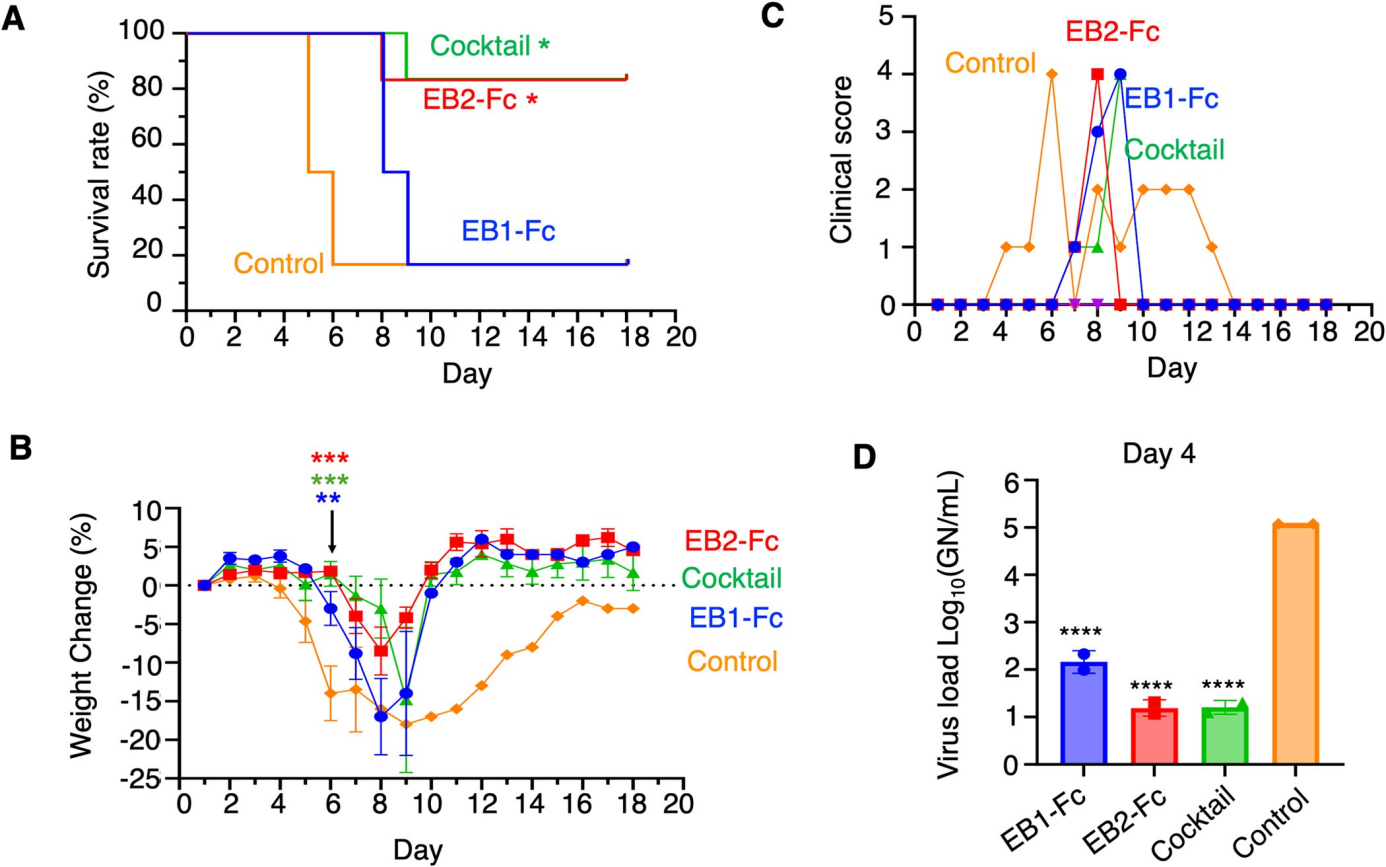
In Vivo Efficacy of Nanosota-EB1-Fc, -EB2-Fc, and Their Cocktail. Groups of 10 mice were challenged with EBOV and then treated with the indicated nanobody, nanobody cocktail, or vehicle alone (control). Each nanobody was administered at a total dose of 50 mg/kg via intraperitoneal (IP) injection. For the cocktail, 25 mg/kg of each nanobody was given. Four animals were used for serum collection and subsequently removed from the study. The remaining 6 animals were monitored for 18 days. **(A)** Survival curves were generated using Kaplan-Meier analysis, and comparisons to the vehicle control were made using the Mantel-Cox test. *P<0.05. **(B)** Weight change for each animal was calculated from its starting weight. Statistical analysis was performed for the data collected on Day 6 using an Ordinary One-Way ANOVA. The average weight change of each experimental group was compared to that of the vehicle control. Error bars on Day 6 represent SEM (n = 3 for control; n = 6 for treatment groups). **P<0.01; ***P<0.001. **(C)** The highest clinical score for each group on each day is displayed. **(D)** Viral loads were measured in mice. Genome copy numbers (GN) were back-calculated from Cq values using a standard curve of synthetic RNA corresponding to the amplicon. Statistical analysis was performed using an Ordinary One-Way ANOVA with multiple comparisons, where each experimental group was compared to the vehicle control. Error bars represent SEM (n = 4). ****P<0.0001.

For in vivo testing, groups of 10 mice were treated 4 hours post-virus challenge with either individual nanobodies or a cocktail of EB1-Fc and EB2-Fc at a dosage of 50 mg/kg via intraperitoneal (IP) injection. Four days post-challenge, 4 mice were euthanized for blood sample collection, while the remaining 6 were monitored for disease progression (Fig 6). In the vehicle-treated group, 5 of 6 mice died by day 6. Similarly, in the EB1-Fc group, 5 of 6 mice also died, but survival was extended by 3 days, with deaths occurring by day 9. In contrast, only 1 of 6 mice died in the EB2-Fc and cocktail groups, with deaths delayed until day 8 and day 9, respectively, indicating significantly improved survival compared to the vehicle control (P = 0.011, Mantel-Cox test) (Fig 6A). In addition to survival, weight loss, a key disease indicator, was also improved. Weight loss onset was delayed by 1, 2, and 3 days in the EB1-Fc, EB2-Fc, and cocktail groups, respectively, compared to the vehicle control, with significant differences across treatments on day 6 (Fig 6B). By day 9, weight loss in the vehicle, EB1-Fc, and cocktail-treated groups converged at 16–19%, whereas the EB2-Fc group showed less weight loss at 10% (Fig 6B). These delays in weight loss were correlated with improved clinical scores based on behavioral and appearance indicators (Fig 6C). Viral loads in serum were measured on day 4, just before the peak serum viral load in this model [43]. Viral genome numbers were quantified by qPCR of RNA extracted from serum. All treatment groups showed significant but similar reductions in viral genome numbers, ranging from 3 x 10^3^ to 10^4^ fold compared to the vehicle group (Fig 6D). Overall, the results demonstrated that EB2-Fc and the cocktail were highly effective in controlling the disease, while EB1-Fc provided modest but significant benefits.

### In vitro stability of Nanosota-EB1 and -EB2

We assessed the in vitro stability of EB1-Fc and EB2-Fc by incubating them at four temperatures (-80°C, 4°C, 25°C, and 37°C) for one week, followed by measuring their remaining GP-ΔM-binding capacity using ELISA (S12 Fig). Using -80°C as the reference, both EB1-Fc and EB2-Fc preserved nearly all of their GP-ΔM-binding capacity at the other three temperatures, including 37°C. As previous EBOV outbreaks occurred in warm regions of Africa with limited access to cold storage, this high in vitro stability offers these two anti-EBOV nanobodies significant advantages, potentially lowering storage and transportation costs.

## Discussion

There remains a clear need for new treatments for filoviruses, despite of the availability of two FDA-approved human antibody drugs (Ebanga and Inmazeb) and several non-FDA-approved human antibodies (ZMapp, FVM04/CA45, MBP134AF, rEBOV-520/548, rEBOV-442/515, 1C3/1C11) for EBOV infections [44–51]. Current antibody-based therapies face significant challenges, including further reducing fatality rates, addressing viral persistence in some survivors, lowering production, transportation, and storage costs, and exploring more accessible administration routes beyond injection. Furthermore, human antibody drugs are not easily adaptable for targeting other EBOV-related filoviruses, such as Bundibugyo ebolavirus (BDBV) and Sudan ebolavirus (SUDV). Nanobodies, with their modular single-domain structure, offer advantages such as easier production, transportation, and storage, the potential for intranasal administration, and the ability to address viral persistence. Notably, nanobodies can be rapidly adapted to target other viral variants or related viruses using a structure-based in vitro evolution strategy that we recently developed [36]. In this study, we developed two nanobodies, Nanosota-EB1 and -EB2, that target EBOV and have potential applications for other viruses within the ebolavirus genus. As the first nanobodies identified to combat authentic filoviruses, Nanosota-EB1 and -EB2 establish a foundation for nanobody-based therapies against filoviruses.

Both Nanosota-EB1 and -EB2 target the EBOV glycoprotein (GP). EB1 binds to and stabilizes the glycan cap in GP1, slowing its protease-mediated cleavage and acting as a moderate inhibitor of EBOV cell entry, a property also observed in glycan cap-binding human antibodies [39]. Furthermore, EB1-Fc has the potential to act as an effector for ADCC and other antiviral immune responses, such as neutrophil activation, phagocytosis, and complement activation, against EBOV infection in vivo [52–54]. However, EB1-Fc exhibited moderate efficacy in the mouse model, only delaying disease onset. This outcome is likely attributed to EB1’s moderate neutralization ability and will be addressed in future research. In contrast, EB2 binds to GP2, targeting the membrane fusion loop, HR1, and the N563 glycan, locking them in their prefusion conformation. Our findings also reveal that EB2 significantly enhances the stability of GP. By stabilizing GP in its prefusion state, EB2 prevents the transition to the post-fusion conformation, introducing a mechanism previously not described for human antibodies. As a result, EB2-Fc demonstrated strong anti-EBOV activity both in vitro and in vivo.

Several human antibodies have been identified that bind to the glycan cap or GP2 of EBOV GP (S13 Fig). Among the three antibodies in the FDA-approved cocktail Inmazeb, REGN-3471 and REGN-3479 target regions similar to those bound by EB1 and EB2, respectively. However, EB1 binds to a unique loop in the glycan cap that differs from the binding site of REGN-3471, though both reduce protease cleavage of GP1’s glycan cap. Additionally, EB2’s interaction with key membrane fusion elements in GP2 provides significant stabilization of GP2, a mechanism not previously reported for human antibodies. Among non-FDA-approved antibodies, EBOV-293 and EBOV-296 also bind to the glycan cap, while KZ52 and ADI-15878 target GP2 [6, 55, 56]. Compared to REGN-3471 and REGN-3479, the epitopes recognized by these non-FDA-approved antibodies differ more but still overlap with those targeted by EB1 and EB2. This overlap is highlighted by the structural clash observed between KZ52 and EB2 when their binding sites are overlaid on GP2 (S13B Fig). Overall, EB1 and EB2 share some overlapping epitopes with several GP-targeting human antibodies, yet our structural and biochemical analyses reveal novel insights into their mechanisms of action.

Although extensive research on anti-EBOV human antibodies has been conducted, this study is among the most comprehensive, encompassing nanobody discovery, structural determination, molecular mechanism elucidation, in vitro antiviral assays, animal challenge studies, and comparisons with human antibodies. Notably, it identifies the first two nanobody inhibitors effective against authentic EBOV. Both nanobodies demonstrate significant promise as anti-EBOV inhibitors, particularly Nanosota-EB2, which exhibits high potency in neutralizing EBOV both in vitro and in mouse models. In addition to their antiviral efficacy, these nanobodies are expected to possess excellent therapeutic qualities, consistent with those previously reported for other nanobodies. While a full evaluation of the therapeutic properties of EB1 and EB2 lies beyond the scope of this study, it highlights that both Fc-tagged nanobodies can be efficiently produced in mammalian cells and exhibit excellent in vitro stability. These properties could help reduce production, storage, and transportation costs. Although neither nanobody is highly effective against two related ebolaviruses, BDBV and SUDV, the small number of residue differences between these viruses suggests that our recently developed structure-guided in vitro evolution approach can adapt these nanobodies to neutralize BDBV and SUDV [36]. This strategy enables the engineering and optimization of existing nanobody inhibitors with known binding epitopes and mechanisms for related viruses, eliminating the need to re-immunize animals to generate new nanobodies from scratch. In summary, Nanosota-EB1 and Nanosota-EB2 establish a strong foundation for the development of nanobody-based therapies against EBOV and related filoviruses.

## Materials and methods

### Ethics statement

This study was performed in strict accordance with the recommendations in the Guide for the Care and Use of Laboratory Animals of the National Institutes of Health. All of the animals were handled according to approved institutional animal care and use committee (IACUC) protocols of Boston University (protocol number: 201900062).

### Cell lines, plasmids and virus

HEK293T and Huh7 cells (American Type Culture Collection (ATCC)) were grown in Dulbecco’s Modified Eagle Medium (DMEM) containing 10% fetal bovine serum, 2 mM L-glutamine, 100 units/mL penicillin, and 100 μg/mL streptomycin. Expi293F cells (ThermoFisher) for protein expression were grown in Expi293 Expression Medium (ThermoFisher). TG1 *E*. *coli* and SS320 *E*. *coli* (Lucigen) for phage display were grown in 2YT medium. No commonly misidentified cell lines were used.

EBOV GP gene (strain Zaire; NCBI Reference Sequence NC_002549.1), BDBV GP gene (GenBank: FJ217161.1), and SUDV GP gene (NCBI Reference Sequence NC_006432.1) were synthesized (GenScript). For pseudovirus packaging, the gene encoding each of the full-length GP proteins was cloned into the pcDNA3.1(+) vector with a C-terminal C9 tag sequence. For protein expressions, the gene encoding each of the GP ectodomains without MLD, named GP-ΔM (residues 1–632, excluding residues 312–463), was cloned into the Lenti-CMV vector (Vigene Biosciences) with a C-terminal foldon trimerization motif sequence followed by a His tag sequence. The gene encoding EBOV sGP (residues1-305) was cloned into the Lenti-CMV vector with a C-terminal His tag sequence. Plasmids encoding Fc-tagged nanobodies were cloned into the Lenti-CMV vector with an N-terminal tPA signal peptide sequence and a C-terminal human IgG1 Fc tag sequence (GenBank: AEV43323.1).

Authentic, replication-competent EBOV (strain H.sapiens-tc/COD/1976/Yambuku-Mayinga) was used to infect Huh7 cells for in vitro assays and interferon-α/β-receptor knockout mice for in vivo assays. Experiments involving infectious EBOV were conducted in approved Biosafety Level 4 laboratories at the National Institute of Allergy and Infectious Diseases (for in vitro assays) and National Emerging Infectious Diseases Laboratories (NEIDL) at Boston University (for in vivo testing).

### Alpaca immunization

Induced nanobody phage display libraries were constructed as previously described [57]. Briefly, an alpaca was immunized subcutaneously in the scapular region with 300 μg EBOV GP-ΔM followed by 3 additional immunizations (2 week intervals) of 150 μg EBOV GP-ΔM.

### Construction of induced nanobody phage display library

Following immunization of the alpaca, blood was drawn, peripheral blood mononuclear cells (PBMCs) were isolated from 35 mL blood and a cDNA library was constructed from the PBMC RNA by reverse transcription using oligo dT primers and Superscript IV reverse transcriptase (ThermoFisher). A nested PCR strategy was used to amplify coding regions of nanobody fragments. The resulting PCR fragments were cloned into a modified pADL22 vector (Antibody Design Labs). The nanobody phage display library with a size of 3 x 10^8^ was constructed following the manufacturer’s protocols (Antibody Design Labs).

### Screening of nanobody phage display library

Screening of nanobody phage display library was conducted as previously described [32]. Briefly, three rounds of bio-panning were performed to identify strong nanobody binders to GP-ΔM. For this purpose, 20 μg of purified GP-ΔM was coated on an immune tube overnight. The coated tube was then blocked with 5% milk and incubated with 500 μl of phages for 1 hour. After washing, the retained phages were eluted and used to infect TG1 *E*. *coli*. The infected TG1 *E*. *coli* were used to amplify phages, which were then employed for the next round of bio-panning. After the third round, the eluted phages were used to infect ss320 *E*. *coli* and then spread onto 2YT agar plates. Single colonies were picked, and nanobody expression was induced using 1 mM IPTG. The supernatants were then subjected to ELISA to identify nanobodies that bound to GP-ΔM.

### Protein expression and purification

His-tagged nanobodies were expressed and purified from the periplasm of ss320 *E*. *coli* as previously described [32]. Briefly, expression of the proteins was induced using 1 mM IPTG. Cell pellets were collected and re-suspended in 15 ml TES buffer (0.2 M Tris pH 8, 0.5 mM EDTA, 0.5 M sucrose), shaken on ice for 1 hour, diluted with 40 ml ¼ TES buffer (TES buffer at ¼ concentration for each component), and then shaken on ice for another hour. The proteins in the supernatant were sequentially purified using a Ni-NTA column and a Superdex200 gel filtration column (Cytiva).

EBOV GP-ΔM, EBOV sGP, and Fc-tagged nanobodies were expressed and purified from mammalian cells as previously described [32, 58]. Briefly, the plasmids encoding each of the above proteins were transiently transfected into the Expi293F cells using polyethylenimine (PEI, Polysciences). 3 days post transfection, the proteins were harvested from the supernatants of cell culture medium. Subsequently, His-tagged EBOV GP-ΔM and EBOV sGP were purified on a Ni-NTA column and purified further on a Superose200 gel filtration column (Cytiva); Fc-tagged nanobodies were purified on a Protein A column and then purified further on a Supedex200 gel filtration column (Cytiva). To produce EBOV GPcl (GP ectodomain with both MLD and the glycan cap removed), 3 mg EBOV GP-ΔM was cleaved with 15 μg thermolysin L (Sigma-Aldrich) at room temperature overnight and then purified on a Superose200 gel filtration column (Cytiva). BDBV GP-ΔM and SUDV GP-ΔM were expressed and purified using the same procedure as EBOV GP-ΔM.

To prepare the complexes of EBOV GP-ΔM and individual nanobodies, EBOV GP-ΔM and each of the His-tagged nanobodies (with the nanobodies in excess) were incubated at room temperature for 1 hour and then were purified on a gel filtration Superose 6 increase 10/300 GL column (Cytiva).

### ELISA

ELISA was conducted to detect the binding between recombinant EBOV GP-ΔM and each of the nanobodies from the supernatant of ss320 *E*. *coli* as previously described [32]. Briefly, ELISA plates were coated with recombinant EBOV GP-ΔM and were then incubated sequentially with the supernatant of ss320 *E*. *coli* (containing HA-tagged nanobodies) and HRP-conjugated anti-HA antibody (1:1,000) (Sigma-Aldrich). ELISA substrate (Invitrogen) was added and then the reactions were stopped using 1N H_2_SO_4_. Absorbances at 450 nm (A_450_) were measured using a Synergy LX Multi-Mode Reader (BioTek).

ELISA was performed to detect the binding between each of the recombinant nanobodies (Nanosota-EB1 and Nanosota-EB2) and each of the recombinant GP proteins (GP-ΔM, GPcl, and sGP from EBOV and GP-ΔM from BDBV and SUDV). The procedure was the same as described above, except that recombinant HA-tagged nanobodies (also His-tagged) replaced the supernatant of ss320 *E*. *coli*.

ELISA was also performed to evaluate the effect of storage conditions on the binding affinity of the Fc-tagged nanobodies to recombinant EBOV GP-ΔM. Briefly, ELISA plates were coated with recombinant EBOV GP-ΔM, followed by the addition of serially diluted nanobody samples. An HRP-conjugated anti-Fc antibody (1:3,000) (Sigma-Aldrich) was then applied. The remaining steps followed the procedure described above.

### Surface plasmon resonance

Surface plasmon resonance (SPR) was carried out to measure the binding affinity between each of the recombinant nanobodies (Nanosota-EB1 and -EB2) and each of the recombinant EBOV GP proteins (GP-ΔM and GPcl) using Biacore S200 system (Cytiva) as previously described [32]. Briefly, one of the recombinant EBOV GP proteins was immobilized on a CM5 sensor chip (Cytiva) through chemical crosslinking. Serial dilutions of one of the His-tagged nanobodies were injected at different concentrations. The resulting data were analyzed using Biacore Evaluation Software (Cytiva).

### Glycan cap cleavage

The cleavage of the GP glycan cap was performed as previously described, with modifications [13, 39]. Briefly, 60 μg of EBOV GP-ΔM complexed with either Nanosota-EB1-His or Nanosota-EB2-His (100 μg) was treated with 0.25 μg of thermolysin L (Sigma-Aldrich) at 37°C for varying durations (5, 15, or 30 minutes). A control sample of 60 μg of EBOV GP-ΔM alone was also prepared. At each time point, aliquots were immediately mixed with SDS-PAGE loading buffer (under reducing conditions) and boiled for 10 minutes to halt the reactions. All samples were then analyzed by SDS-PAGE and Coomassie blue staining. To further confirm the cleavage of the GP glycan cap, Western blot analysis was performed following an extended incubation period. In this procedure, 60 μg of EBOV GP-ΔM complexed with either Nanosota-EB1-Fc or Nanosota-EB2-Fc (100 μg) was treated with 0.25 μg of thermolysin L (Sigma-Aldrich) at 37°C for different durations (15, 30, or 60 minutes). At each time point, aliquots were immediately mixed with SDS-PAGE loading buffer (under non-reducing conditions) and boiled for 10 minutes to stop the reactions. GP-ΔM was detected using an anti-His tag antibody (Sigma-Aldrich, 1:1,000).

### Pseudovirus cell entry assay

The EBOV pseudovirus entry assay was conducted to measure the neutralizing potencies of nanobodies against EBOV pseudoviruses, as previously described [30]. Briefly, EBOV pseudoviruses were produced by co-transfecting HEK293T cells with a pcDNA3.1(+) plasmid encoding the full-length EBOV GP, a lentiviral packaging plasmid psPAX2, and a reporter plasmid plenti-CMV-luc. After 72 hours, the pseudoviruses were collected, incubated with each of the nanobodies at different concentrations at 37°C for 1 hour, and then used to infect Huh7 cells. After an additional 48 hours, the cells were lysed. Portions of the cell lysates were transferred to new plates, a luciferase substrate was added, and Relative Light Units (RLUs) were measured using an EnSpire plate reader (PerkinElmer). The efficacy of each nanobody was determined and expressed as the concentration required to inhibit pseudovirus entry by 50% (IC_50_).

BDBV and SUDV pseudovirus entry assays were performed following the same procedure as the EBOV pseudovirus entry assay, except that BDBV GP and SUDV GP were used in place of EBOV GP, respectively.

### Authentic EBOV neutralization assay

The neutralizing potency of the nanobodies was evaluated using authentic EBOV. Nanobodies were diluted to specified concentrations in cell culture medium and incubated with the virus for 60 minutes. Infection levels were assessed using an immunofluorescence assay, where Hoechst staining (ThermoFisher) was used to visualize cell nuclei, with nuclei counts serving as a proxy for cell counts. The efficacy of each nanobody was determined by calculating the concentration required to reduce infected cell counts by 50% (IC_50_) compared to the virus-exposed control group without nanobody treatment.

### Mouse efficacy study

The efficacy of the nanobody treatments was evaluated in an interferon-α/β-receptor knockout (IFNAR-KO) mouse model (strain B6(Cg)-Ifnar1tm1.2Ees/J; Jackson Laboratories) as previously described [59]. All animal procedures were conducted by the Animal Studies Core at the National Emerging Infectious Disease Laboratory at Boston University under biosafety level 4 (ABSL-4) conditions. Briefly, 8- to 12-week-old mice were randomly assigned to groups of 10, with equal numbers of males and females in each group. All mice were challenged with 100 PFU of EBOV in a 0.1 mL volume of PBS buffer via intraperitoneal (IP) injection on day 0. Treatments, dissolved in Dulbecco’s phosphate-buffered saline (PBS, Gibco), were administered once via IP injection 4 hours post-infection. Group 1 received Nanosota-EB1-Fc at 50 mg/kg, group 2 received Nanosota-EB2-Fc at 50 mg/kg, group 3 received a combination of Nanosota-EB1-Fc and EB2-Fc at a total dosage of 50 mg/kg (25 mg/kg of each nanobody), and group 4 received PBS alone. All animals were monitored daily for survival, weight, and clinical signs, with observations conducted twice daily during the critical phase (days 3–10). Scoring criteria for humane endpoints were previously defined based on correlative analysis of factors associated with disease outcomes, including eye appearance, general appearance, responsiveness, and body weight loss (>11%). Animals meeting three of the four criteria during two consecutive observation periods were humanely euthanized following American Veterinary Medical Association (AVMA) and Association for Assessment and Accreditation of Laboratory Animal Care (AAALAC) guidelines. Animals experiencing 20% body weight loss or agonal states were also euthanized.

On day 4, four animals (two males and two females) per group were randomly selected, euthanized, and serum samples collected. These animals were excluded from survival analyses. The remaining animals continued in the study under the same conditions until day 18, at which point the study concluded.

Day 4 was selected for viral load measurement based on a previous study indicating that viral titers had not yet reached the plateau phase on this day [43], making it an ideal time point for comparisons. Furthermore, untreated mice began to succumb by day 5, making later time points unsuitable for measuring viral load. Serum samples collected on day 4 were inactivated using Trizol LS (Invitrogen) and analyzed for viral genome copy numbers (GN). More specifically, total RNA was extracted using the Direct-zol RNA Miniprep Kit (Zymo Research) following the manufacturer’s instructions. RNA concentration and quality were assessed using a NanoDrop spectrophotometer (NanoDrop Technologies). EBOV-specific primers (IDT, 5’-CATGCGTACCAGGGAGATTAC-3’ and 5’-ACTCCATCACGCTTCTTGAC-3’) and a probe (IDT, 5’-/56 FAM/TCAAGTATT/ZEN/TGGAAGGGCACGGGT/3IABkFQ/-3’) were used in a reverse transcriptase quantitative PCR (RT-qPCR) assay. The assay was performed using the Luna One-Step Universal Probe RT-qPCR Kit (New England Biolabs) on a BioRad instrument, with analysis conducted using BioRad Maestro software. A standard curve was generated using an EBOV RNA standard (transcribed from a PCR-derived EBOV NP fragment template) through 10-fold serial dilutions in water. Each sample, including standards, was run in duplicate along with a non-targeting control. The average values from duplicates were used to calculate genome copy numbers in the serum samples using the standard curve.

### Thermal stability assay

To evaluate the impact of nanobodies on the thermal stability of EBOV GP, differential scanning fluorimetry (DSF) experiments were conducted as previously described, with modifications [60]. Briefly, 20 μg of EBOV GP-ΔM was incubated with 4 μg of Nanosota-EB1-His or Nanosota-EB2-His separately at room temperature for 30 minutes. Each sample was then mixed with 1.25 μl of Protein Thermal Shift Dye (ThermoFisher) diluted in 10 μl of PBS buffer in a 96-well MicroAmp optical qPCR plate. For the control group, 20 μg of EBOV GP-ΔM was incubated with Protein Thermal Shift Dye alone. Measurements were performed using a PCR instrument (Applied Biosystems) with a temperature ramp from 25 to 99°C at a rate of 0.05°C/s. Data were collected using QuantStudio real-time PCR software. The negative first derivative of fluorescence was plotted against temperature, and the melting temperature (Tm) was determined as the peak point in the first derivative curve.

### Cryo-EM data collection

4 μl of purified complexes of EBOV GP-ΔM and individual nanobody at ~1.25 μM were used for grid preparation. Each of the complexes was applied to freshly glow-discharged Quantifoil R1.2/1.3 300-mesh copper grids (EM Sciences) and blotted for 4 seconds at 22°C under 100% chamber humidity and plunge-frozen in liquid ethane using a Vitrobot Mark IV (FEI). Cryo-EM data were collected using Latitude-S (Gatan) equipped with a K3 direct electron detector and with a Biocontinuum energy filter (Gatan). For the GP-ΔM /Nanosota-EB1 complex, movies were collected at a nominal magnification of 81,000x (corresponding to 1.1 Å per pixel). For the GP-ΔM /Nanosota-EB2 complex, movies were collected at a nominal magnification of 130,000x (corresponding to 0.664 Å per pixel). Statistics of cryo-EM data collection are summarized in S1 Table.

### Cryo-EM data processing, model building and refinement

Cryo-EM data were processed using cryoSPARC v3.3.2 [61], and the procedures are outlined in S4 and S5 Figs. Briefly, dose-fractionated movies were subjected to Patch motion correction with MotionCor2 [62] and Patch CTF estimation with CTFFIND-4.1.13 [63]. Particles were then picked using the Blob picker in cryoSPARC v3.3.2 and subjected to the Remove Duplicate Particles tool. Junk particles were removed through three rounds of 2D classifications. Particles from the good 2D classes were used for ab-initio reconstruction of four maps. The initial models were set as the starting references for heterogeneous refinement (3D classification). The good 3D classes were then subjected to further homogeneous, non-uniform, and CTF refinements to generate the final maps, applying C1 and C3 symmetry for the GP-ΔM/Nanosota-EB1 complex and GP-ΔM/Nanosota-EB2 complex, respectively. Map resolutions were determined using gold-standard Fourier shell correlation (FSC) at 0.143 between the two half-maps.

Initial model building of the EBOV GP/nanobody complex was performed in Coot-0.8.9 [64] using 5JQ7 as the starting model. The initial model of each nanobody was predicted using SWISS-MODEL (https://swissmodel.expasy.org/) and then fitted into the density map. Several rounds of refinement in Phenix-1.16 [65] and manual building in Coot-0.8.9 were performed until the final reliable models were obtained. Model and map statistics are summarized in S1 Table. Figures were generated using UCSF Chimera X v0.93 [66]. EBOV GP and nanobody contact residues were analyzed using LigPlot, and the EBOV GP and nanobody buried interfaces were analyzed using PDBePISA (https://www.ebi.ac.uk/pdbe/pisa/). Representative structures in density are presented in S6 Fig.

### Statistical analysis

For the mouse efficacy study, GraphPad Prism (version 10.3.0) was used for data analysis and statistical assessments. Survivorship curves were generated using the Kaplan-Meier method, and comparisons with the vehicle control were performed using the Mantel-Cox test. Percent weight change for each individual animal was calculated relative to its starting weight. P-values for percent weight change were calculated using an Ordinary One-Way ANOVA on data collected on day 6, comparing each experimental group to the vehicle control. Clinical scores were recorded as the highest score for each group on each day. Viral loads were analyzed using standard methods, with genome numbers (GN) expressed per milliliter. GN/mL values were log-transformed, and P-values were calculated using an Ordinary One-Way ANOVA with multiple comparisons on data collected on day 4, comparing each experimental group to the vehicle control.

For all other statistical analyses, unpaired two-tailed Student’s *t*-tests were performed. Please refer to the Figure Legends and Supporting Information Legends for details.

## Disclaimer Note

The views and conclusions contained in this document are those of the authors and should not be interpreted as necessarily representing the official policies, either expressed or implied, of the U.S. Department of Health and Human Services or of the institutions and companies affiliated with the authors, nor does mention of trade names, commercial products, or organizations imply endorsement by the U.S. Government.

## Supporting information

S1 Fig Structural overview of EBOV GP. (A) The overall structure of the EBOV GP ectodomain (PDB: 5JQ3). (B) Schematic representations of four EBOV GP variants: GP ectodomain, GP-ΔM, GPcl, and sGP. GP1 is the receptor-binding subunit, and GP2 is the membrane-fusion subunit. RBS: receptor-binding site. MLD: mucin-like domain. HR1: heptad repeat 1. HR2: heptad repeat 2. SP: signal peptide.
(TIF)

S2 Fig Surface Plasmon Resonance (SPR) Sensorgrams. **(A)** SPR sensorgrams of binding kinetics between Nanosota-EB1-His and EBOV GP-ΔM. **(B)** No binding was detected between Nanosota-EB1-His and EBOV GPcl. **(C)** SPR sensorgrams of binding kinetics between Nanosota-EB2-His and EBOV GP-ΔM. **(D)** SPR sensorgrams of binding kinetics between Nanosota-EB2-His and EBOV GPcl. K_d_, *k*_*on*_, and *k*_*off*_ values are labeled.
(TIF)

S3 Fig Efficacy of His-tagged nanobodies in neutralizing EBOV pseudoviruses. The assay was conducted as described in Fig 1C.
(TIF)

S4 Fig Flow Chart of Cryo-EM Image Processing and Map Reconstruction for the Structures of EBOV GP-ΔM Complexed with Nanosota-EB1. Representative raw cryo-EM images and 2D classes of the complex are presented. 3D refinement using all the particles in good 3D classes generated a 3.07 Å map. The final maps, half-map FSC curves, angular distribution plot, and accompanying local resolution illustrations are enclosed in the dashed black box.
(TIF)

S5 Fig Flow Chart of Cryo-EM Image Processing and Map Reconstruction for the Structures of EBOV GP-ΔM Complexed with Nanosota-EB2. Representative raw cryo-EM images and 2D classes of the complex are presented. 3D refinement using all the particles in good 3D classes generated a 3.36 Å map. The final maps, half-map FSC curves, angular distribution plot, and accompanying local resolution illustrations are enclosed in the dashed black box.
(TIF)

S6 Fig Cryo-EM Densities of Selected Regions in the Structures of EBOV GP-ΔM Complexed with Nanosota-EB1 or Nanosota-EB2. **(A-B)** Cryo-EM densities of the interface between Nanosota-EB1 and EBOV GP. **(C-H)** Cryo-EM densities of the interface between Nanosota-EB2 and EBOV GP. Nanobodies are colored in blue. Different parts of EBOV GP are colored differently. Contact residues are shown as sticks.
(TIF)

S7 Fig Nanosota-EB1 Binds to a Cryptic Epitope on the EBOV GP Glycan Cap. **(A)** Left panel: Structure of the EBOV GP glycan cap without a bound ligand (PDB: 9BSV). The glycan cap appears disordered due to its flexibility. Right panel: Structure of the EBOV GP glycan cap bound to Nanosota-EB1. Binding by Nanosota-EB1 stabilizes the glycan cap, making it visible. **(B)** Structure of the EBOV GP glycan cap bound to the human antibody REGN-3470 (PDB: 7TN9). Structural motifs of the glycan cap are labeled. The bound REGN-3470 is depicted as a gray oval. **(C)** Structure of the EBOV GP glycan cap bound to Nanosota-EB1. Nanosota-EB1 disrupts the β18 strand and interacts with the inner β17 strand, revealing part of the β17/18 loop. The bound Nanosota-EB1 is illustrated as a blue oval. **(D)** Comparison of the structures of the EBOV GP glycan cap bound to the human antibody EBOV-548 (PDB: 6UYE) and the glycan cap bound to Nanosota-EB1. Both the human antibody and Nanosota-EB1 target the β17 strand but approach it from different orientations.
(TIF)

S8 Fig Superimposition of N563 Glycan from Different Structures. Three structures of EBOV GP (PDBs: 9BSU, 9BSV, and 7TN9) are superimposed by their HR1 region. The N563 glycan from each of the three structures is colored green, orange, and magenta, respectively.
(TIF)

S9 Fig Impacts of Nanosota-EB1 and Nanosota-EB2 on Thermostability of EBOV GP-ΔM and GPcl at Different pHs. The positive first derivatives are plotted against temperature. The melting temperatures (Tm) at the peak point in the first derivative curve are shown at the top of each valley. pH values are labeled for each panel.
(TIF)

S10 Fig Glycan Cap Cleavage Assay to Evaluate the Effect of Nanosota-EB2 on the Protease Sensitivity of the Glycan Cap. **(A)** The assay was conducted using SDS-PAGE under reducing conditions and Coomassie blue staining. Nanosota-EB2 presence had no obvious effect on the thermolysin L cleavage of GP-ΔM. **(B)** The assay was performed using Western blot to detect the His tag on GP-ΔM under non-reducing conditions. Nanosota-EB2 presence again had no obvious effect on the thermolysin L cleavage of GP-ΔM. Each of the above experiments was performed three times, yielding consistent results.
(TIF)

S11 Fig Quantification of Western blot results for the glycan cap proteolysis assay. ImageJ (version 1.53a) was used to quantify the GP-ΔM monomer bands from the Western blot data in Fig 4D and its two replicates for EB1-Fc **(A)** and in S10B Fig and its two replicates for EB2-Fc **(B)**. Unpaired two-tailed Student’s *t*-tests were conducted to compare the treatment condition and the control condition at each time point (n = 3). **p<0.01. n.s.: not significant.
(TIF)

S12 Fig In Vitro Stability of Nanosota-EB1-Fc and Nanosota-EB2-Fc. ELISA was performed to assess the effect of storage conditions on the binding affinity of the Fc-tagged nanobodies to recombinant EBOV GP-ΔM.
(TIF)

S13 Fig Comparison of the Epitopes on EBOV GP Recognized by Nanobodies (Nanosota-EB1 and Nanosota-EB2) and Human Antibodies. **(A)** Epitopes on the glycan cap of GP1. PDB IDs of human antibodies are indicated. Epitope residues were analyzed using LigPlot+ v.2.2 and are displayed on the GP monomer in surface mode, with different colors representing different antibodies: blue for Nanosota-EB1, cyan for REGN-3470, green for EBOV-293, and magenta for EBOV-296. **(B)** Epitopes on GP2. PDB IDs of human antibodies are indicated. Epitope residues are shown in surface mode on the two monomers of the GP trimer, with colors distinguishing antibodies: blue for Nanosota-EB2, cyan for REGN-3479, green for KZ52, and magenta for ADI-15878. An overlay of KZ52 and Nanosota-EB2 on the same GP2 structure reveals a clash, suggesting overlapping binding epitopes.
(TIF)

S1 Table Cryo-EM Data Collection, Refinement, and Validation Statistics. (PDF)

S2 Table Detailed Interactions Between EBOV GP and Nanosota-EB1. (PDF)

S3 Table Detailed Interactions Between EBOV GP and Nanosota-EB2. (PDF)
