# Supplementary figures and images for: Discovery of Nanosota-EB1 and -EB2 as Novel Nanobody Inhibitors Against Ebola Virus Infection
Source: PLoS Pathog. 2024 Dec 23;20(12):e1012817. doi: 10.1371/journal.ppat.1012817 (PMC11723632; doi:10.1371/journal.ppat.1012817)

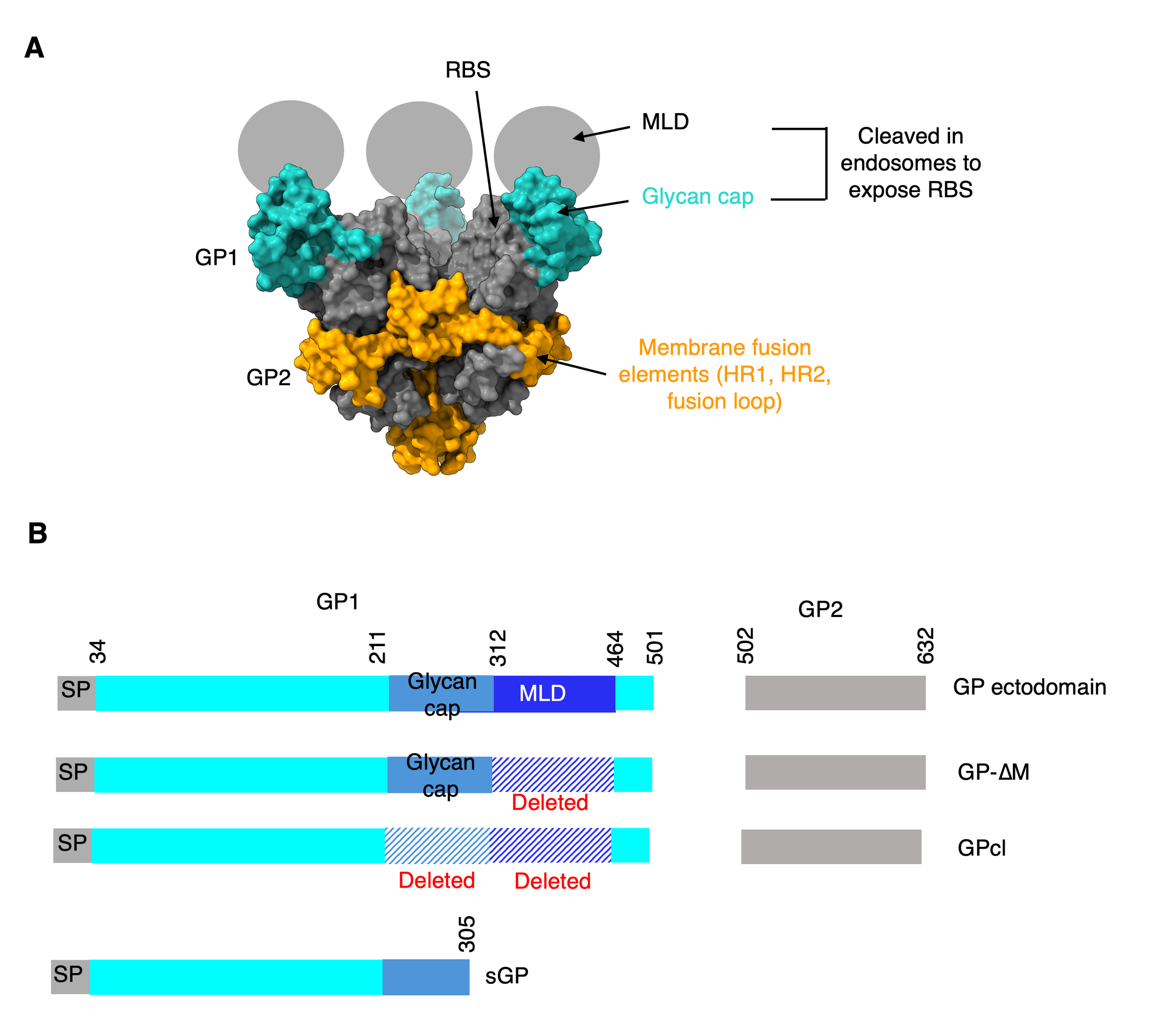

Supplement: S1 Fig — (A) The overall structure of the EBOV GP ectodomain (PDB: 5JQ3). (B) Schematic representations of four EBOV GP variants: GP ectodomain, GP-ΔM, GPcl, and sGP. GP1 is the receptor-binding subunit, and GP2 is the membrane-fusion subunit. RBS: receptor-binding site. MLD: mucin-like domain. HR1: heptad repeat 1. HR2: heptad repeat 2. SP: signal peptide. (TIF) [file ppat.1012817.s001.tif]

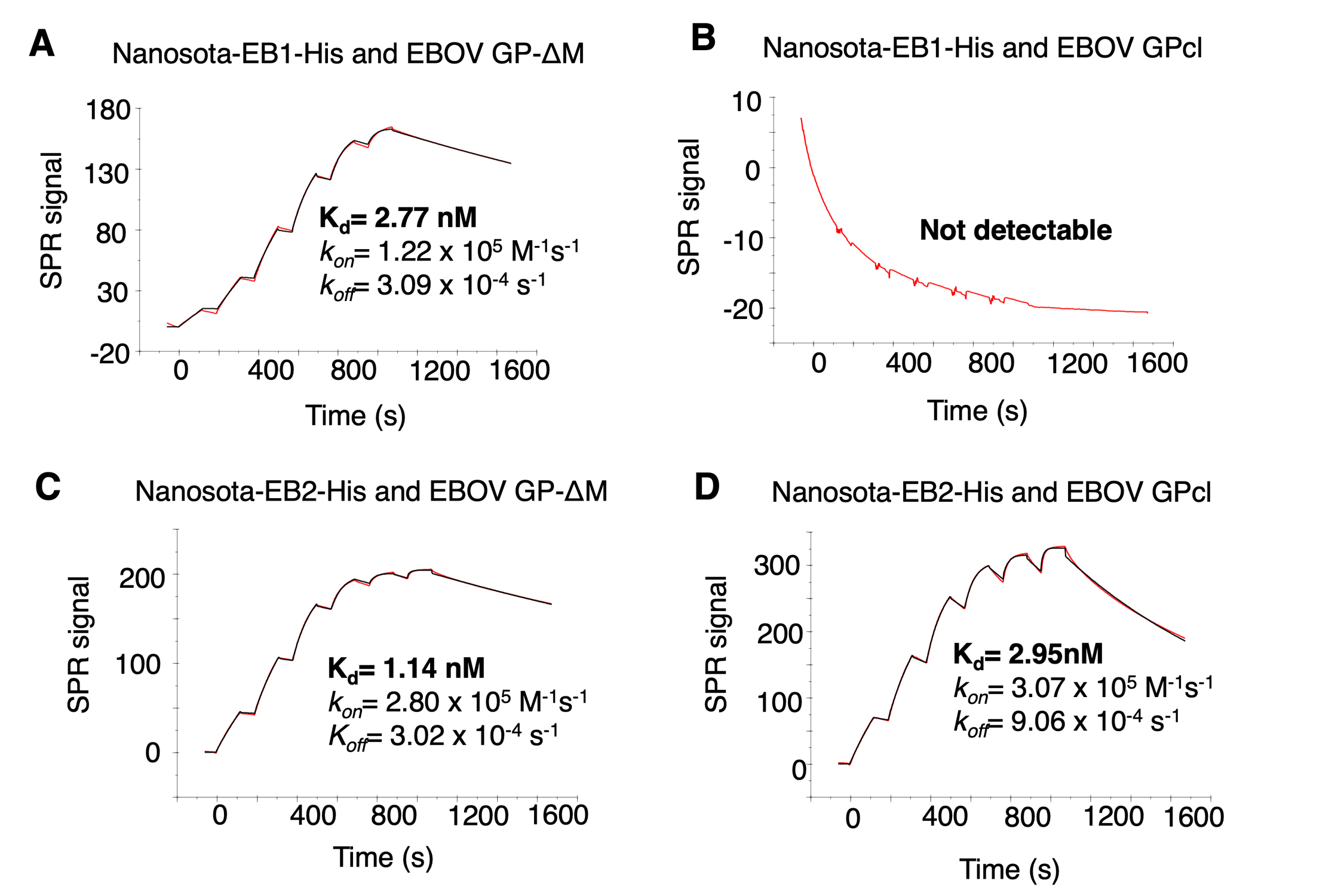

Supplement: S2 Fig — (A) SPR sensorgrams of binding kinetics between Nanosota-EB1-His and EBOV GP-ΔM. (B) No binding was detected between Nanosota-EB1-His and EBOV GPcl. (C) SPR sensorgrams of binding kinetics between Nanosota-EB2-His and EBOV GP-ΔM. (D) SPR sensorgrams of binding kinetics between Nanosota-EB2-His and EBOV GPcl. Kd, kon, and koff values are labeled. (TIF) [file ppat.1012817.s002.tif]

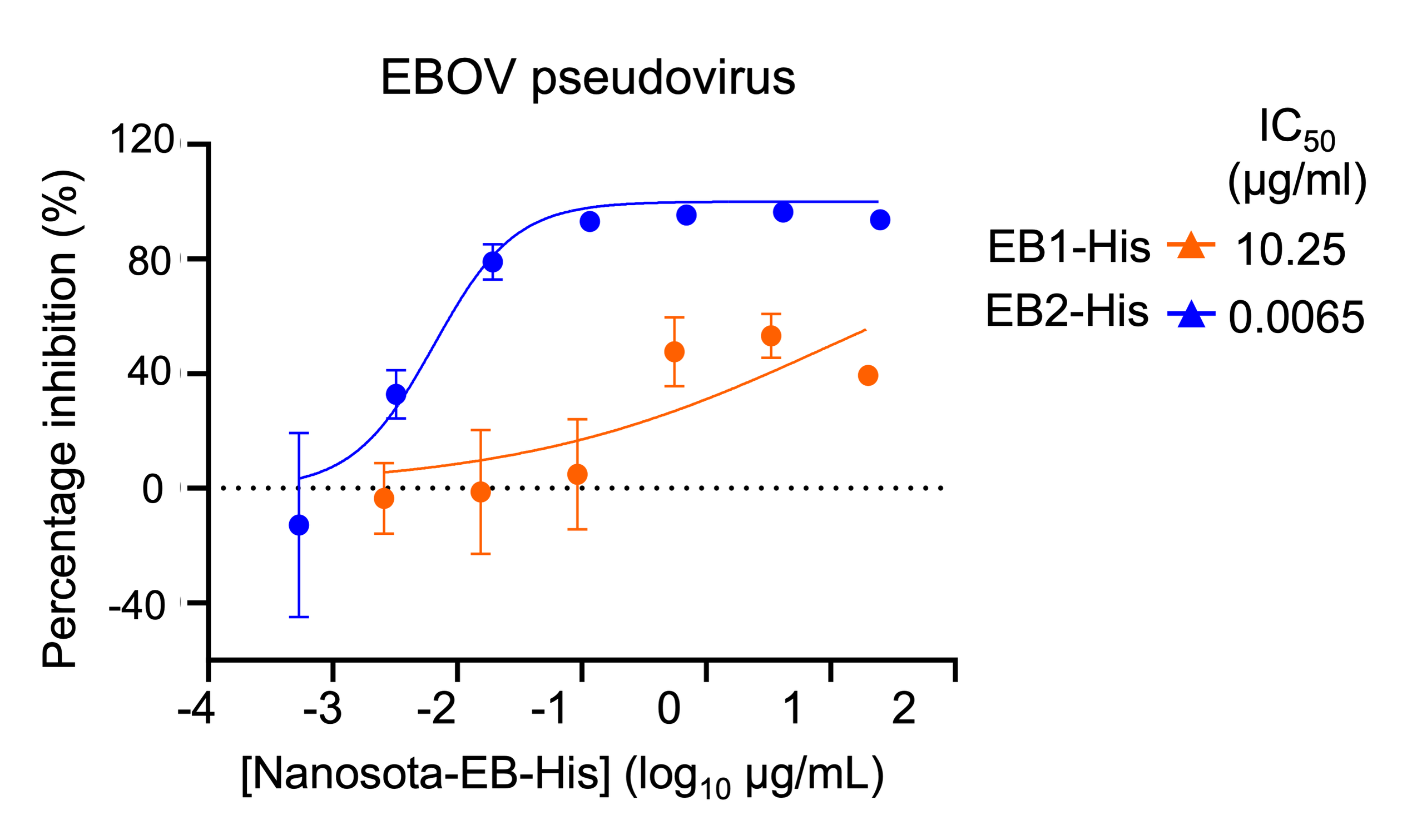

Supplement: S3 Fig — The assay was conducted as described in Fig 1C. (TIF) [file ppat.1012817.s003.tif]

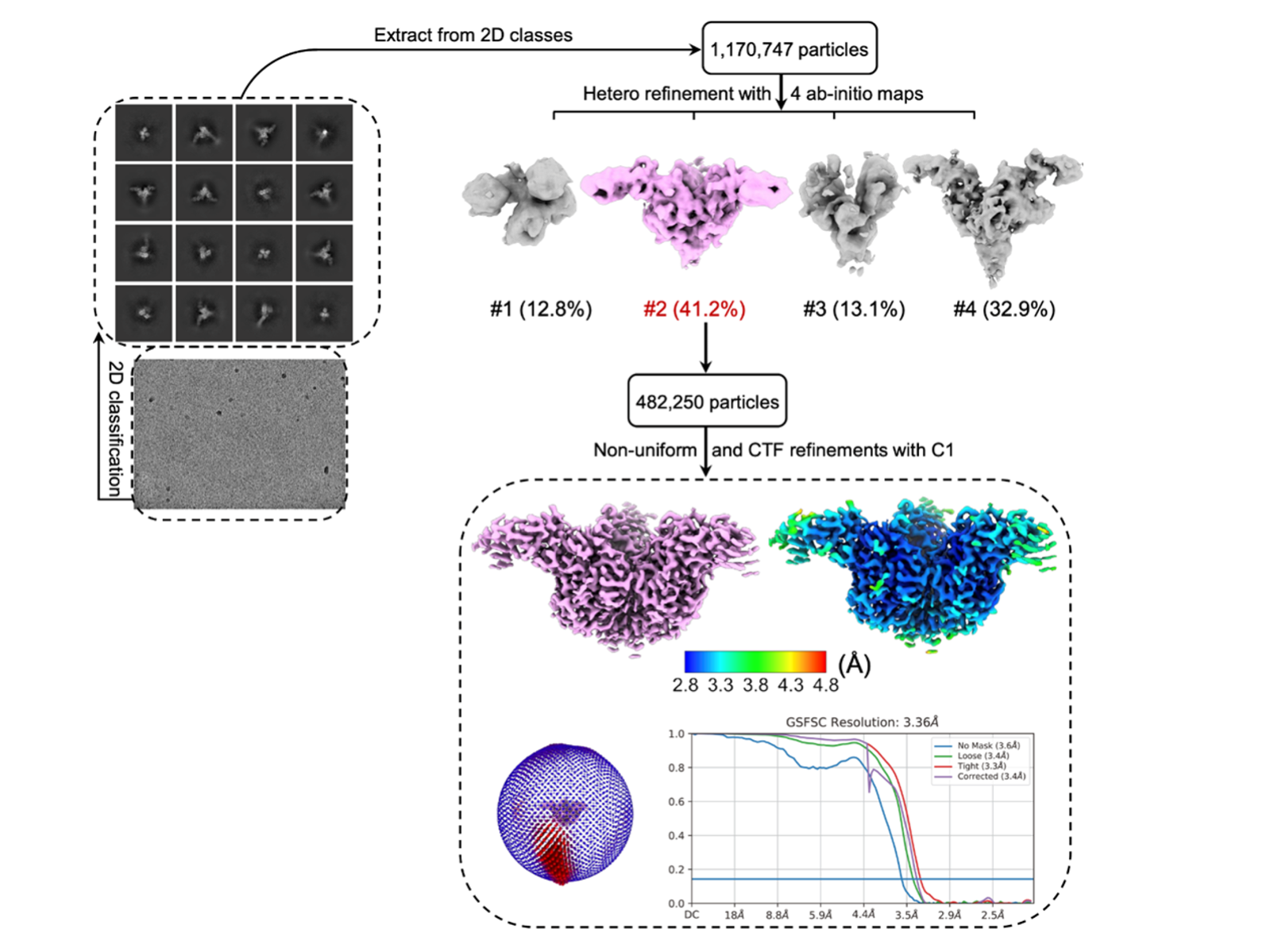

Supplement: S4 Fig — Representative raw cryo-EM images and 2D classes of the complex are presented. 3D refinement using all the particles in good 3D classes generated a 3.07 Å map. The final maps, half-map FSC curves, angular distribution plot, and accompanying local resolution illustrations are enclosed in the dashed black box. (TIF) [file ppat.1012817.s004.tif]

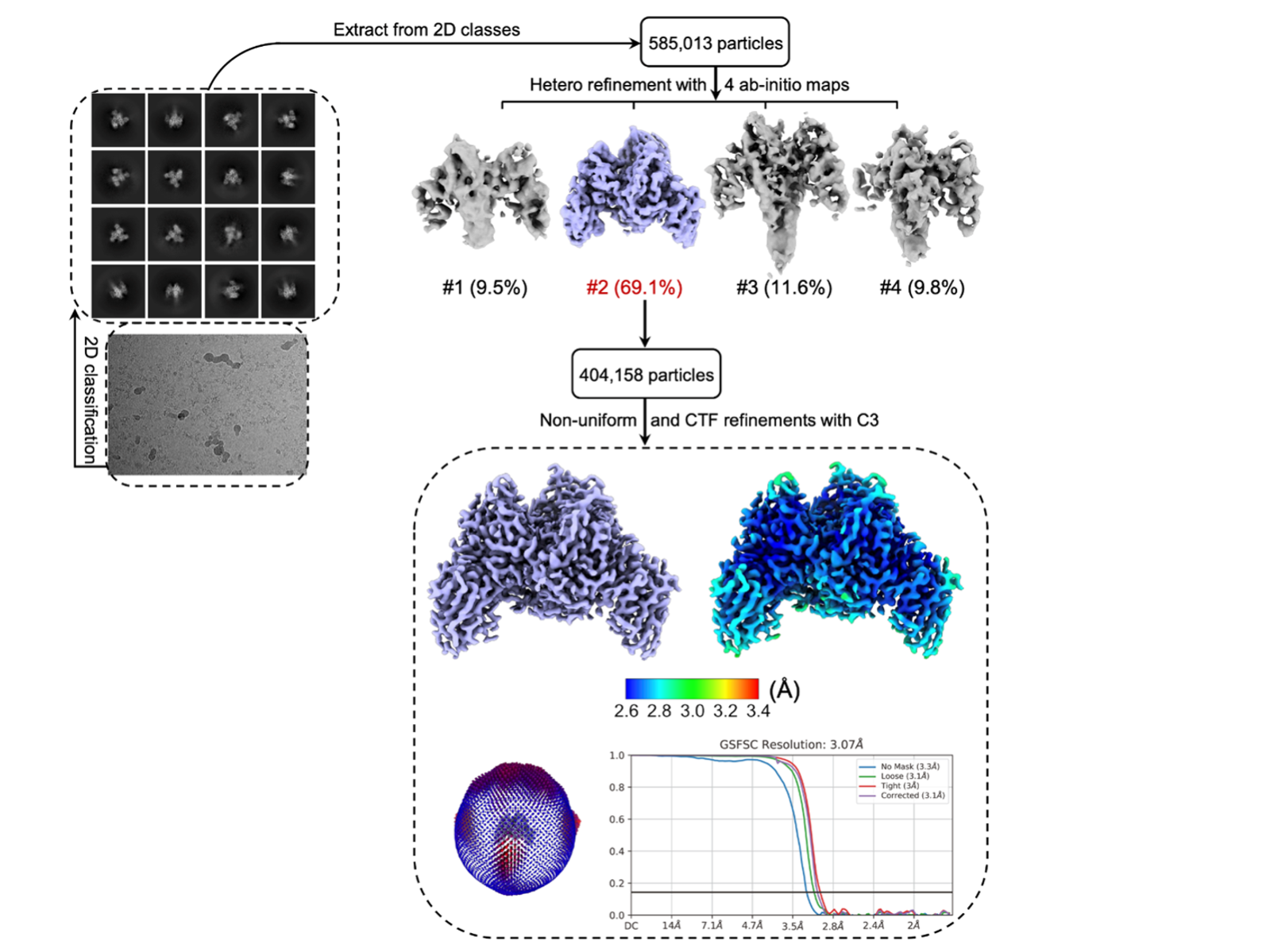

Supplement: S5 Fig — Representative raw cryo-EM images and 2D classes of the complex are presented. 3D refinement using all the particles in good 3D classes generated a 3.36 Å map. The final maps, half-map FSC curves, angular distribution plot, and accompanying local resolution illustrations are enclosed in the dashed black box. (TIF) [file ppat.1012817.s005.tif]

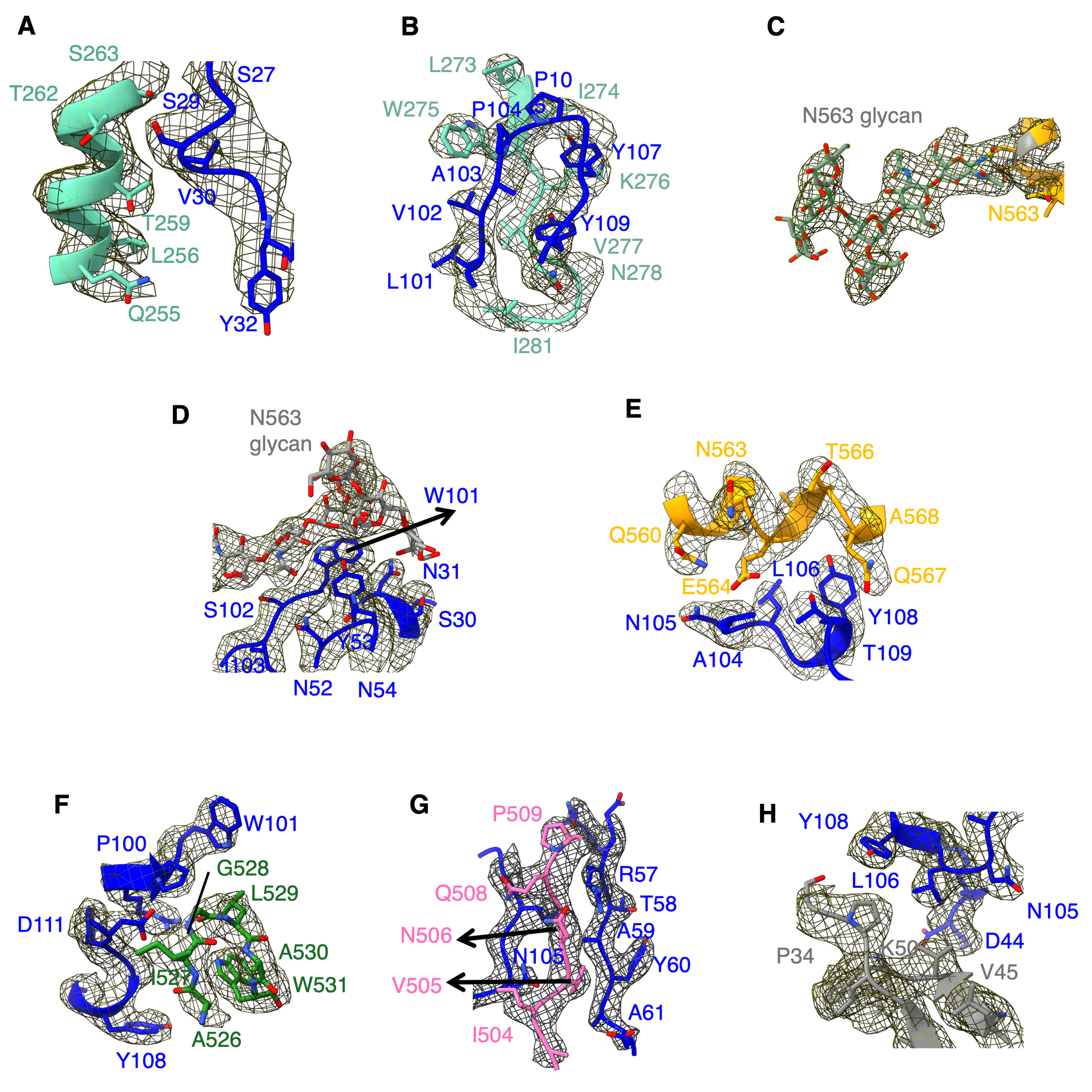

Supplement: S6 Fig — (A-B) Cryo-EM densities of the interface between Nanosota-EB1 and EBOV GP. (C-H) Cryo-EM densities of the interface between Nanosota-EB2 and EBOV GP. Nanobodies are colored in blue. Different parts of EBOV GP are colored differently. Contact residues are shown as sticks. (TIF) [file ppat.1012817.s006.tif]

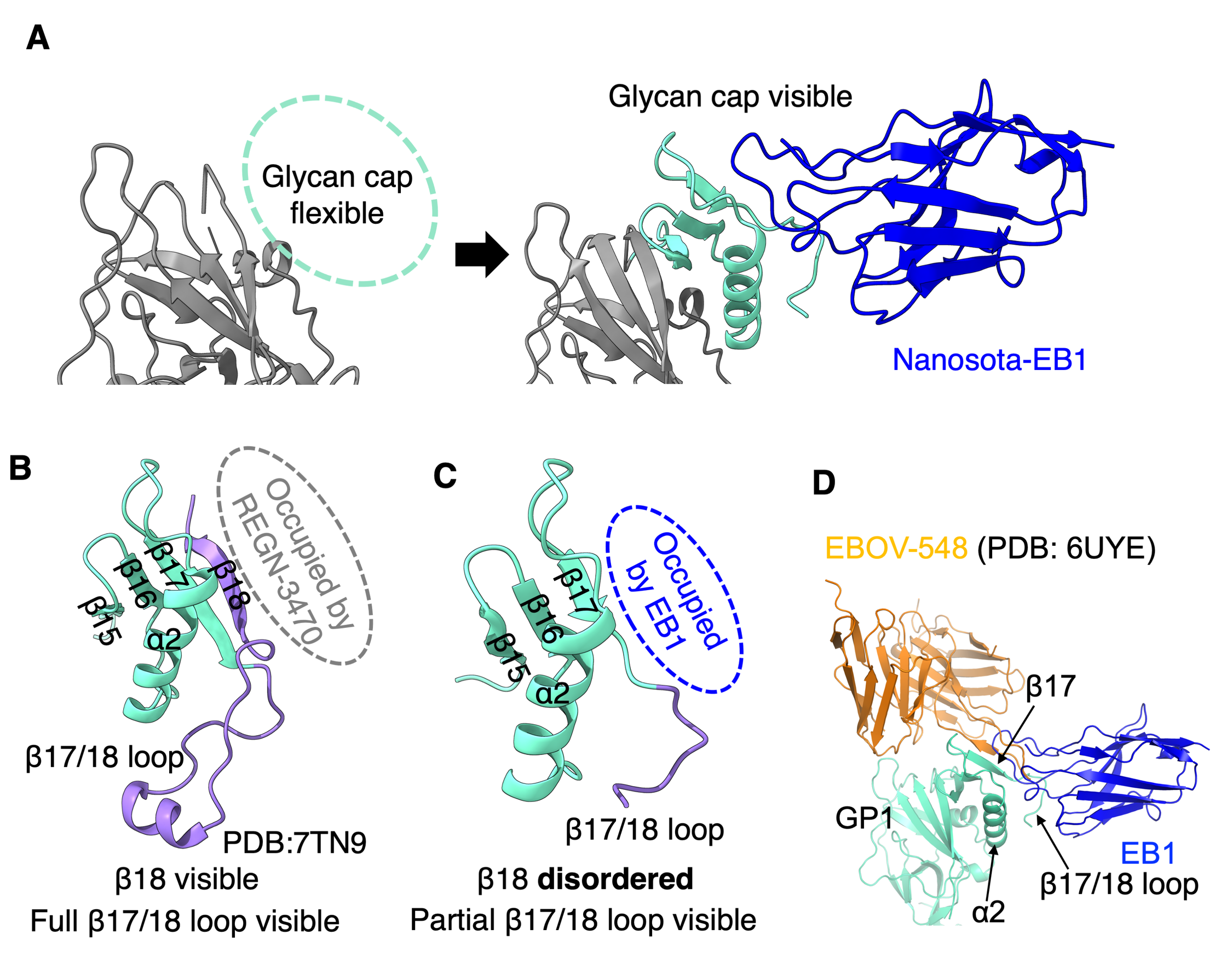

Supplement: S7 Fig — (A) Left panel: Structure of the EBOV GP glycan cap without a bound ligand (PDB: 9BSV). The glycan cap appears disordered due to its flexibility. Right panel: Structure of the EBOV GP glycan cap bound to Nanosota-EB1. Binding by Nanosota-EB1 stabilizes the glycan cap, making it visible. (B) Structure of the EBOV GP glycan cap bound to the human antibody REGN-3470 (PDB: 7TN9). Structural motifs of the glycan cap are labeled. The bound REGN-3470 is depicted as a gray oval. (C) Structure of the EBOV GP glycan cap bound to Nanosota-EB1. Nanosota-EB1 disrupts the β18 strand and interacts with the inner β17 strand, revealing part of the β17/18 loop. The bound Nanosota-EB1 is illustrated as a blue oval. (D) Comparison of the structures of the EBOV GP glycan cap bound to the human antibody EBOV-548 (PDB: 6UYE) and the glycan cap bound to Nanosota-EB1. Both the human antibody and Nanosota-EB1 target the β17 strand but approach it from different orientations. (TIF) [file ppat.1012817.s007.tif]

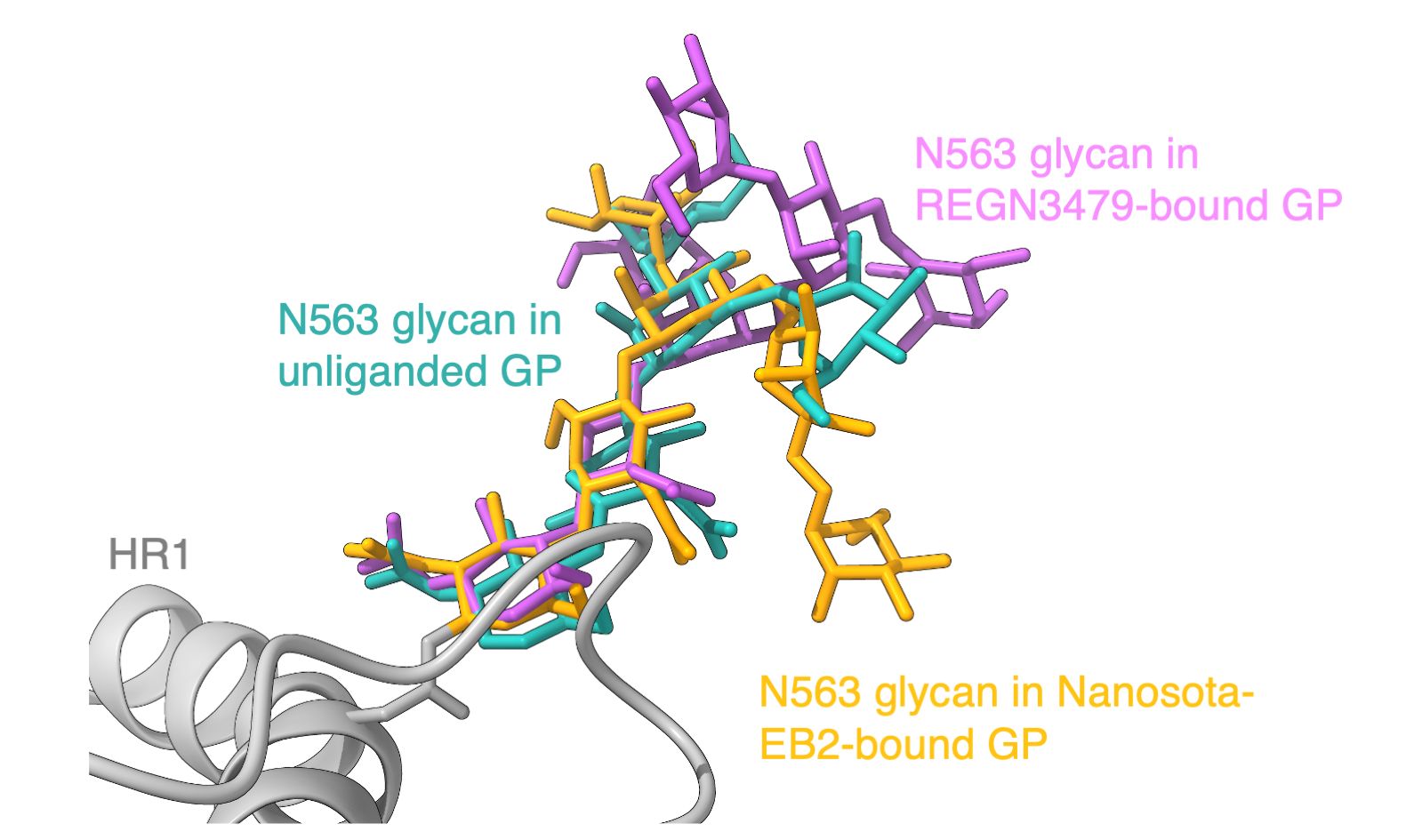

Supplement: S8 Fig — Three structures of EBOV GP (PDBs: 9BSU, 9BSV, and 7TN9) are superimposed by their HR1 region. The N563 glycan from each of the three structures is colored green, orange, and magenta, respectively. (TIF) [file ppat.1012817.s008.tif]

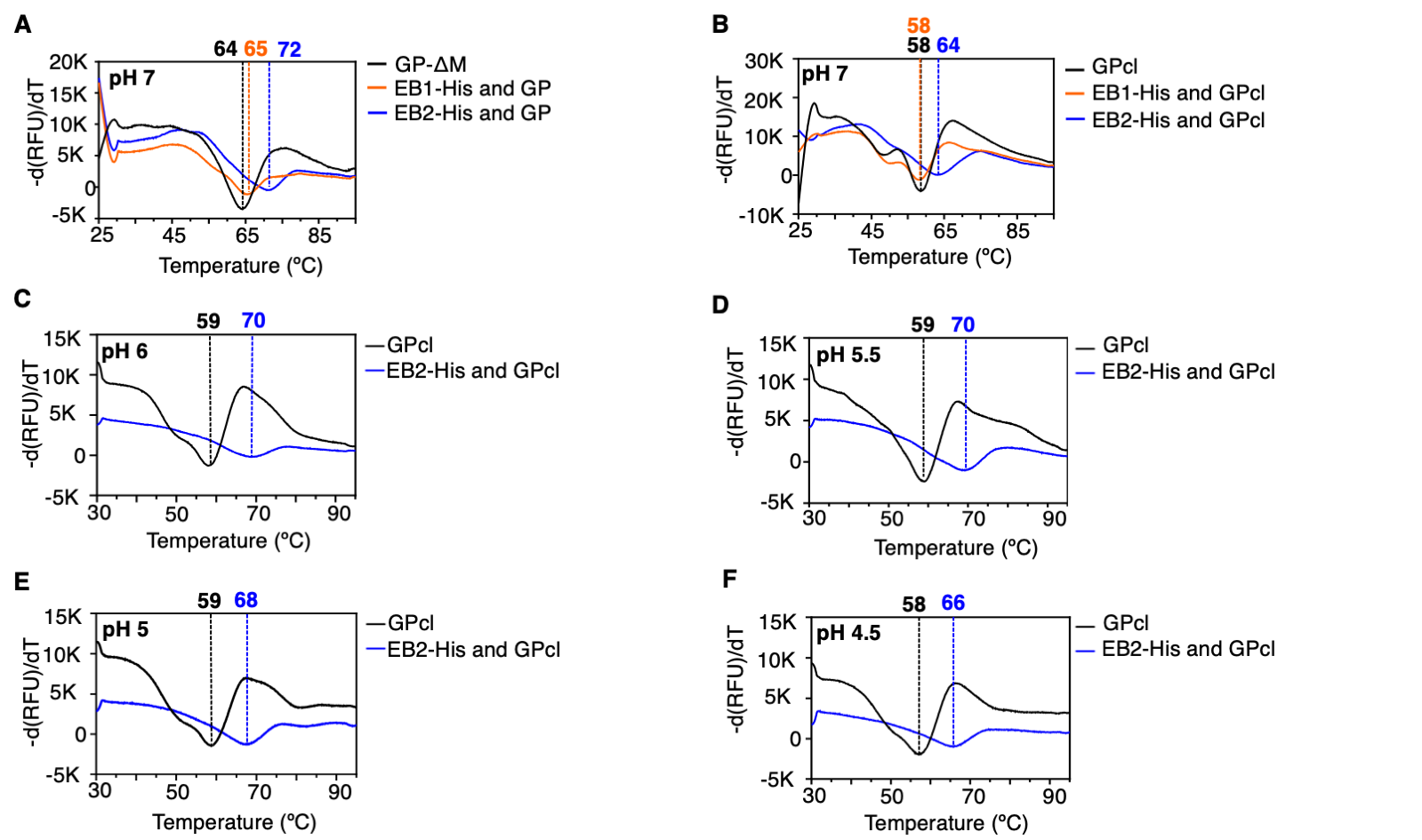

Supplement: S9 Fig — The positive first derivatives are plotted against temperature. The melting temperatures (Tm) at the peak point in the first derivative curve are shown at the top of each valley. pH values are labeled for each panel. (TIF) [file ppat.1012817.s009.tif]

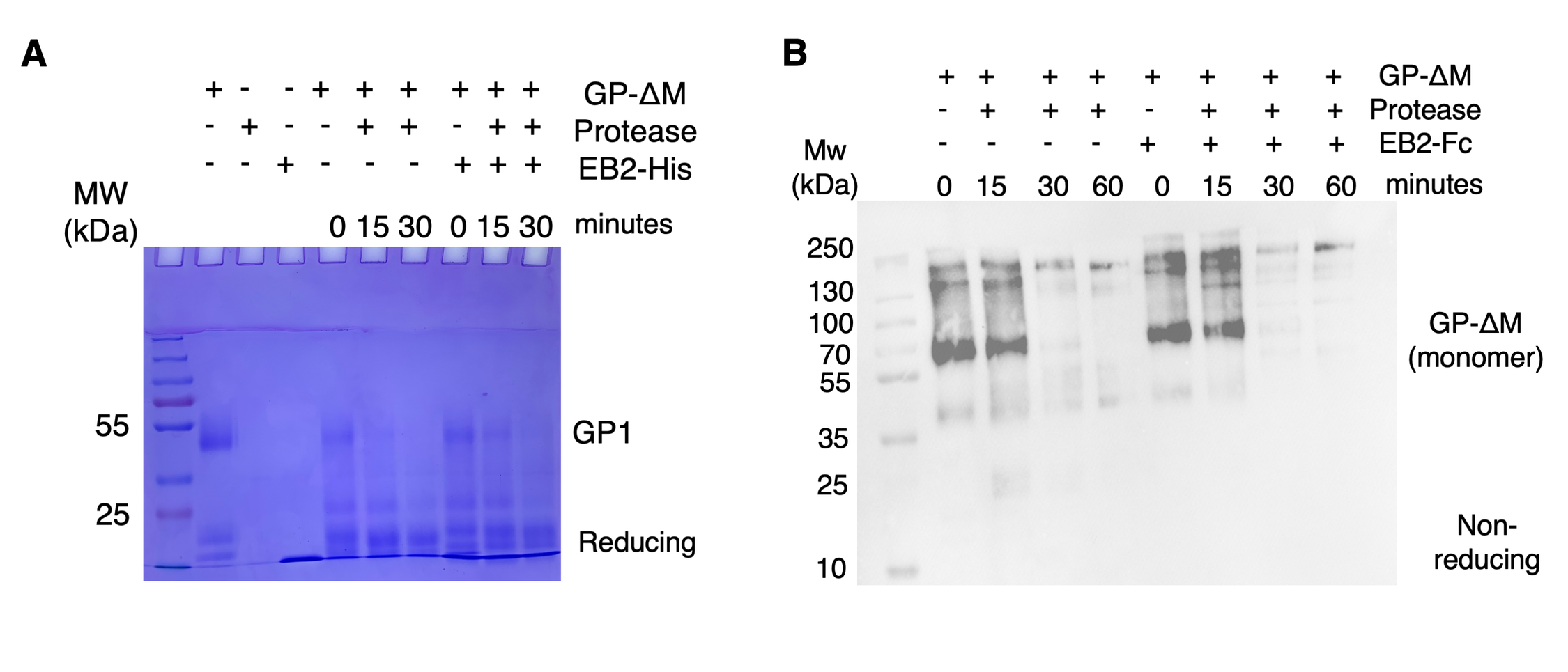

Supplement: S10 Fig — (A) The assay was conducted using SDS-PAGE under reducing conditions and Coomassie blue staining. Nanosota-EB2 presence had no obvious effect on the thermolysin L cleavage of GP-ΔM. (B) The assay was performed using Western blot to detect the His tag on GP-ΔM under non-reducing conditions. Nanosota-EB2 presence again had no obvious effect on the thermolysin L cleavage of GP-ΔM. Each of the above experiments was performed three times, yielding consistent results. (TIF) [file ppat.1012817.s010.tif]

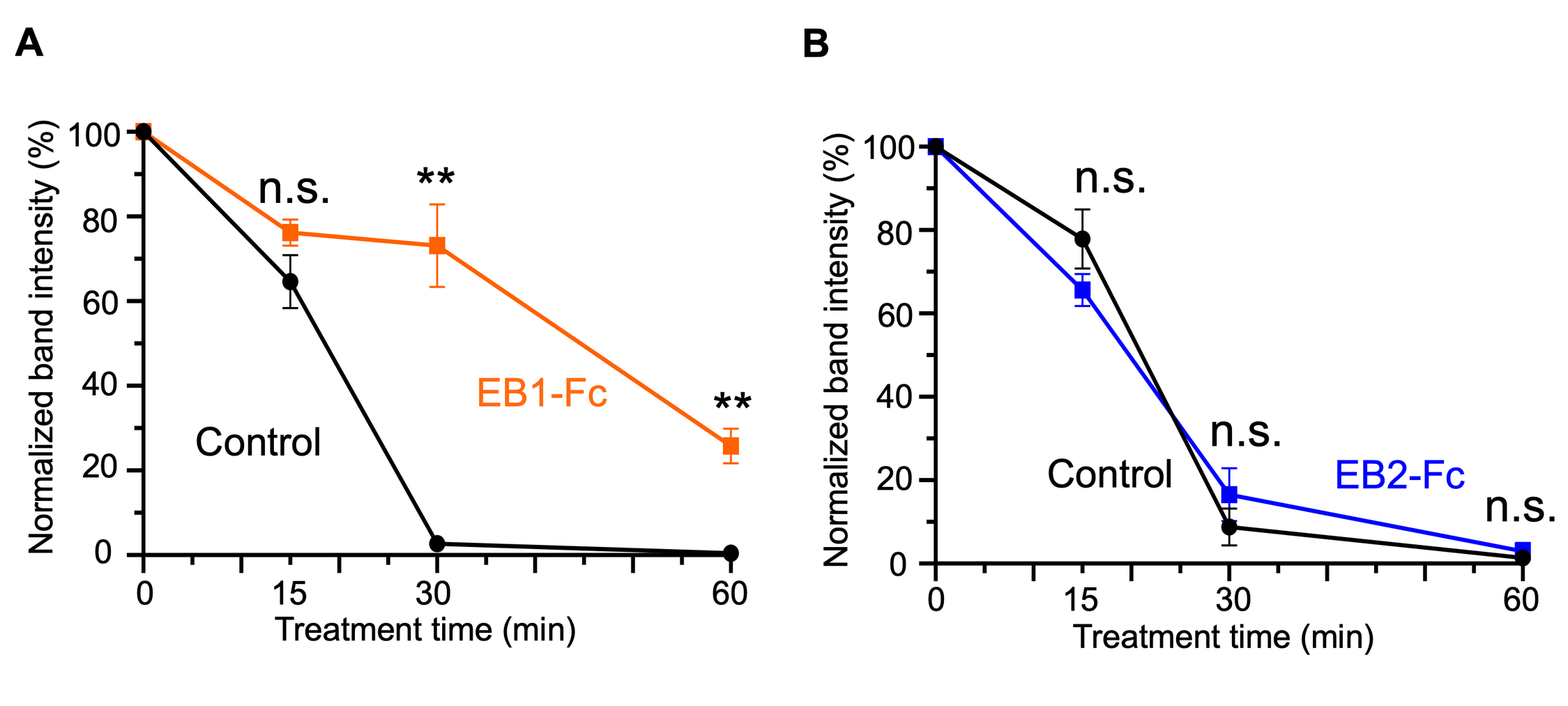

Supplement: S11 Fig — ImageJ (version 1.53a) was used to quantify the GP-ΔM monomer bands from the Western blot data in Fig 4D and its two replicates for EB1-Fc (A) and in S10B Fig and its two replicates for EB2-Fc (B). Unpaired two-tailed Student’s t-tests were conducted to compare the treatment condition and the control condition at each time point (n = 3). **p<0.01. n.s.: not significant. (TIF) [file ppat.1012817.s011.tif]

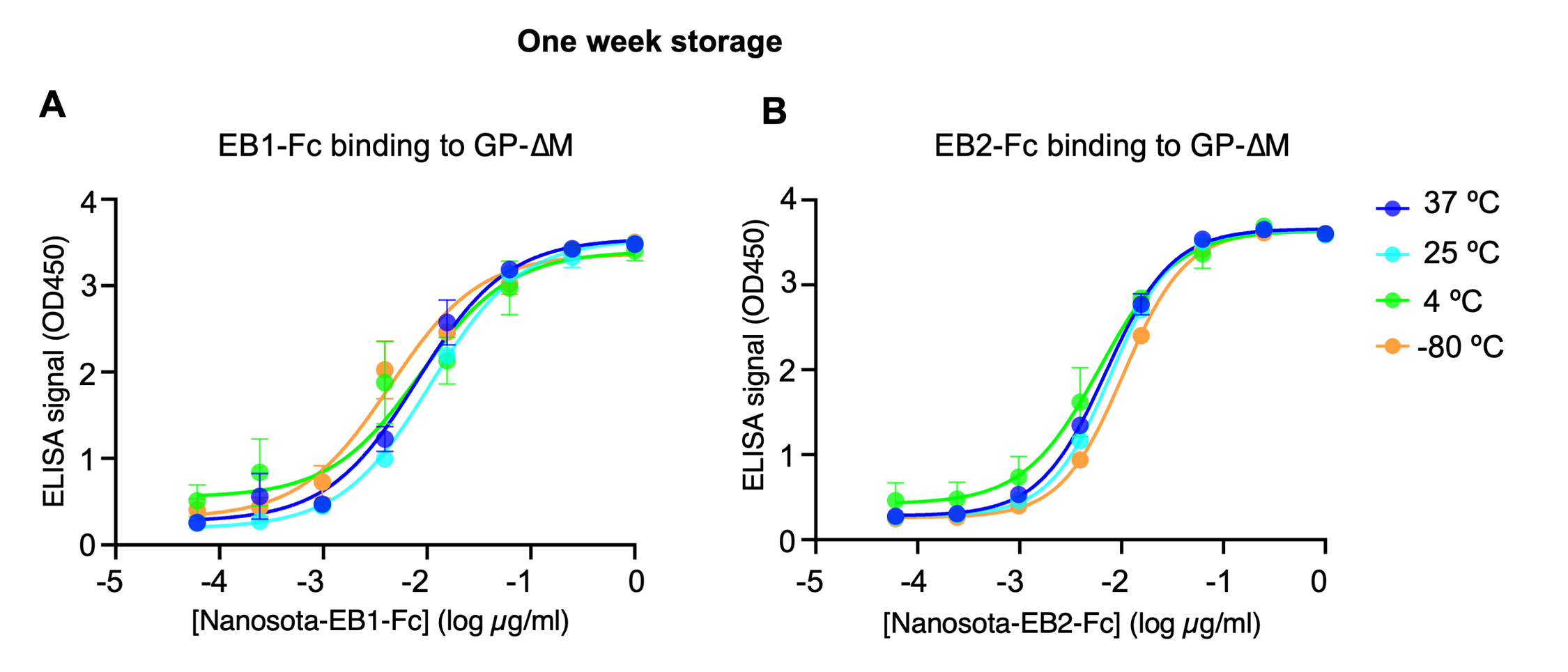

Supplement: S12 Fig — ELISA was performed to assess the effect of storage conditions on the binding affinity of the Fc-tagged nanobodies to recombinant EBOV GP-ΔM. (TIF) [file ppat.1012817.s012.tif]

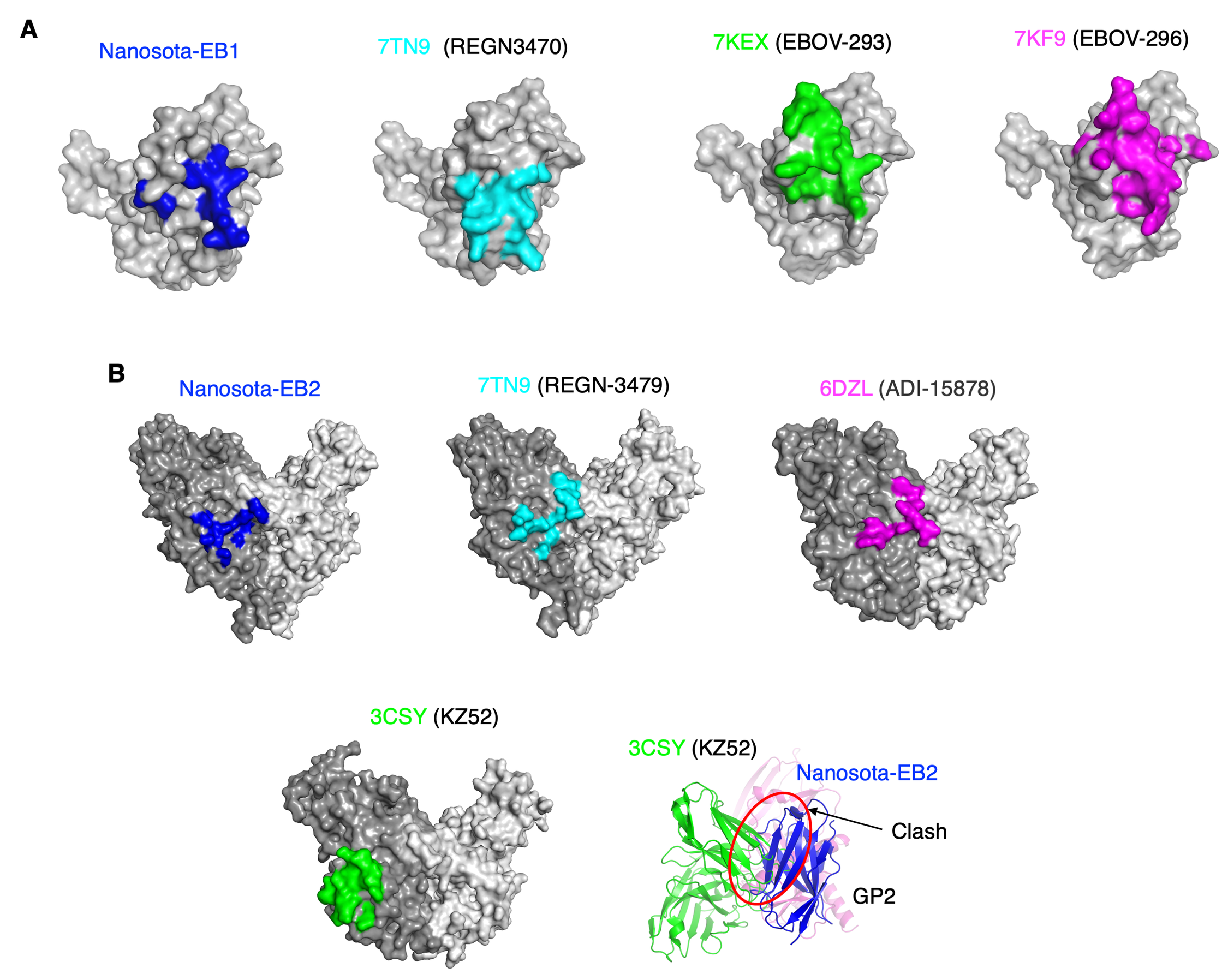

Supplement: S13 Fig — (A) Epitopes on the glycan cap of GP1. PDB IDs of human antibodies are indicated. Epitope residues were analyzed using LigPlot+ v.2.2 and are displayed on the GP monomer in surface mode, with different colors representing different antibodies: blue for Nanosota-EB1, cyan for REGN-3470, green for EBOV-293, and magenta for EBOV-296. (B) Epitopes on GP2. PDB IDs of human antibodies are indicated. Epitope residues are shown in surface mode on the two monomers of the GP trimer, with colors distinguishing antibodies: blue for Nanosota-EB2, cyan for REGN-3479, green for KZ52, and magenta for ADI-15878. An overlay of KZ52 and Nanosota-EB2 on the same GP2 structure reveals a clash, suggesting overlapping binding epitopes. (TIF) [file ppat.1012817.s013.tif]
