# Supplementary material for: Discovery of Nanosota-EB1 and -EB2 as Novel Nanobody Inhibitors Against Ebola Virus Infection
Source: PLoS Pathog. 2024 Dec 23;20(12):e1012817. doi: 10.1371/journal.ppat.1012817 (PMC11723632; doi:10.1371/journal.ppat.1012817)
Supplement: S1 Table — (PDF) [file ppat.1012817.s014.pdf]

Table S1: Cryo-EM Data Collection, Refinement, and Validation Statistics

	EBOV GP / Nanosota-EB1 (EMD-44872) (PDB 9BSU)	EBOV GP / Nanosota-EB2 (EMD-44873) (PDB 9BSV)
**Data collection and processing**		
Magnification	81,000	130,000
Voltage (kV)	300	300
Electron exposure (e–/Å^2)	45.00	50.00
Defocus range (μm)	-1.0 ~ -2.0	-1.0 ~ -2.0
Pixel size (Å)	1.1	0.664
Symmetry imposed	C1	C3
Initial particle images (no.)	1,170,747	585,013
Final particle images (no.)	482,250	404,158
Map resolution (Å)	3.4	3.1
FSC threshold	0.143	0.143
Map resolution range (Å)	2.8–4.8	2.6-3.4
**Refinement**		
Initial model used (PDB code)	5JQ7	5JQ7
Model resolution (Å)	3.5	3.2
FSC threshold	0.5	0.5
Model resolution range (Å)	32.3-3.3	26.5-3.0
Map sharpening *B* factor (Å^2)	-119.2	-144.7
Model composition		
Non-hydrogen atoms	10227	9882
Protein residues	1287	1248
Ligands	16	21
B factors (Å^2)		
Protein	45.26	108.00
Nucleotide		
Ligand	39.39	120.44
R.m.s. deviations		
Bond lengths (Å)	0.004	0.005
Bond angles (°)	0.777	0.704
Validation		
MolProbity score	1.89	1.77
Clashscore	8.78	7.83
Poor rotamers (%)	0.19	0.69
Ramachandran plot		
Favored (%)	93.75	95.10
Allowed (%)	6.02	4.17
Disallowed (%)	0.24	0.74
