# Supplementary material for: Discovery of Nanosota-EB1 and -EB2 as Novel Nanobody Inhibitors Against Ebola Virus Infection
Source: PLoS Pathog. 2024 Dec 23;20(12):e1012817. doi: 10.1371/journal.ppat.1012817 (PMC11723632; doi:10.1371/journal.ppat.1012817)
Supplement: S2 Table — (PDF) [file ppat.1012817.s015.pdf]

Table S2: Detailed Interactions Between EBOV GP and Nanosota-EB1

	Nanosota-EB1 binding footprint		Corresponding residues in other ebolaviruses	
Domain	Nanobody residues interacting with EBOV GP	EBOV GP residues	BDBV GP residues	SUDV GP residues
GP1-Glycan cap, α2	Leu101	Leu256	Conserved	Conserved
GP1-Glycan cap, α2	Val30	Thr259	Conserved	Conserved
GP1-Glycan cap, α2	Ser29	Thr262	Conserved	Leu262
GP1-Glycan cap, α2	Ser29, Ser27	Ser263	Asn263	His263
GP1-Glycan cap, β17	Pro105	Leu273	Conserved	Conserved
GP1-Glycan cap, β17	Tyr107	Ile274	Conserved	Conserved
GP1-Glycan cap, β17	Pro104, Val102, Ala103	Trp275	Conserved	Conserved
GP1-Glycan cap, β17	Tyr109, Tyr107, Val102	Lys276	Conserved	Thr276
GP1-Glycan cap, β17	Tyr109, Leu101	Val277	Conserved	Leu277
GP1-Glycan cap, β17	Leu101, Tyr109	Asn278	Conserved	Asp278
GP1-Glycan cap, β17	Tyr32	Ile281	Val281	Conserved

Residues that differ between EBOV and other ebolaviruses are labeled in red.
