# Supplementary material for: Discovery of Nanosota-EB1 and -EB2 as Novel Nanobody Inhibitors Against Ebola Virus Infection
Source: PLoS Pathog. 2024 Dec 23;20(12):e1012817. doi: 10.1371/journal.ppat.1012817 (PMC11723632; doi:10.1371/journal.ppat.1012817)
Supplement: S3 Table — (PDF) [file ppat.1012817.s016.pdf]

Table S3: Detailed Interactions Between EBOV GP and Nanosota-EB2

	Nanosota-EB2 binding footprint		Corresponding residues in other ebolaviruses	
Domain	Nanobody residues interacting with EBOV GP	EBOV GP residues	BDBV GP residues	SUDV GP residues
Monomer A (GP2, fusion loop)	Asp111	Gly528	Conserved	Conserved
Monomer A (GP2, fusion loop)	Pro100	Leu529	Conserved	Ile529
Monomer B (GP1, β1-β2 strands)	Leu106	Pro34	Conserved	Conserved
Monomer B (GP1, β1-β2 strands)	Leu106	Val45	Conserved	Conserved
Monomer B (GP2, HR1A)	Ala104, Asn105, Tyr108	Glu564	Conserved	Conserved
Monomer B (GP2, HR1A)	Tyr108, Thr109	Gln567	Conserved	Conserved
Monomer B (GP2, HR1A)	Tyr108	Ala568	Conserved	Conserved
Monomer B (GP2, HR1A, ASN563 glycan)	Ser30, Asn31, Asn52, Tyr53, Asn54, Arg109, Trp101, Ser102, Ile103	ASN563 Glycan	Conserved	Conserved
Monomer B (GP2, N terminus)	Tyr60, Asn105	Val505	Lys505	Thr505
Monomer B (GP2, N terminus)	Arg57	Asn506	Arg506	Lys506
Monomer B (GP2, N terminus)	Arg57, Ile103	Ala507	Thr507	Conserved
Monomer B (GP2, N terminus)	Arg57	Gln508	Conserved	Thr508

Residues that differ between EBOV and other ebolaviruses are labeled in red.
